# Limitations associated with transcranial direct current stimulation for enhancement: considerations of performance tradeoffs in active-duty Soldiers

**DOI:** 10.3389/fnhum.2024.1444450

**Published:** 2024-07-26

**Authors:** Michelle J. Duffy, Kathryn A. Feltman, Amanda M. Kelley, Ryan Mackie

**Affiliations:** ^1^U.S. Army Aeromedical Research Laboratory, Fort Novosel, AL, United States; ^2^Oak Ridge Institute for Science and Education, Oak Ridge, TN, United States

**Keywords:** working memory, attention, executive function, tDCS, active-duty Soldiers

## Abstract

**Introduction:**

Transcranial direct current stimulation (tDCS) is a non-invasive brain stimulation method, popular due to its low cost, ease-of-application, and portability. As such, it has gained traction in examining its potential for cognitive enhancement in a diverse range of populations, including active-duty military. However, current literature presents mixed results regarding its efficacy and limited evaluations of possible undesirable side-effects (such as degradation to cognitive processes).

**Methods:**

To further examine its potential for enhancing cognition, a double-blind, randomized, sham-controlled, within-subjects design, was used to evaluate both online active-anodal and -cathodal on several cognitive tasks administered. Potential undesirable side effects related to mood, sleepiness, and cognitive performance, were also assessed. Active tDCS was applied for 30 min, using 2 mA, to the left dorsolateral prefrontal cortex with an extracephalic reference placed on the contralateral arm of 27 (14 males) active-duty Soldiers.

**Results:**

We report mixed results. Specifically, we found improvements in sustained attention (active-anodal) for males in reaction time (*p* = 0.024, η*p*^2^ = 0.16) and for sensitivity index in females (*p* = 0.013, η*p*^2^ = 0.18). In addition, we found faster reaction time (*p* = 0.034, η*p*^2^ = 0.15) and increased accuracy (*p* = 0.029, η*p*^2^ = 0.16) associated with executive function (active-anodal and -cathodal), and worsened working memory performance (active-cathodal; *p* = 0.008, η*p*^2^ = 0.18). Additionally, we found increased risk-taking with active-anodal (*p* = 0.001, η*p*^2^ = 0.33).

**Discussion:**

tDCS may hold promise as a method for cognitive enhancement, as evidenced by our findings related to sustained attention and executive function. However, we caution that further study is required to better understand additional parameters and limitations that may explain results, as our study only focused on anode vs. cathode stimulation. Risk-taking was examined secondary to our main interests which warrants further experimental investigation isolating potential tradeoffs that may be associated with tDCS simulation.

## 1 Introduction

Transcranial direct current stimulation (tDCS) is a form of non-invasive brain stimulation. It has been the subject of investigation in a wide range of studies for both its potential therapeutic properties in pathological populations, as well as for potentially enhancing cognition in healthy populations (Doruk et al., 2014; Rassovsky et al., 2018; Brauer et al., 2021). One subset of the healthy population where improved cognitive processing is of great interest is amongst military members. Individuals within the military are required to make quick decisions and execute tasks accurately and quickly. Whether tDCS can assist in improving these types of cognitive processes is of interest to military stakeholders. In addition to potential performance enhancements, performance trade-offs should also be considered. McKinley et al. (2012) expressed the need to evaluate potential side effects of transcranial electrical stimulation (TES) and more specifically tDCS, whereas the cost of enhancing one cognitive function (e.g., working memory) may come at the cost of inhibiting or “hurting” another (e.g., impulsivity). According to the theory of zero-sum gains, an area of cognitive enhancement or achievement must be matched by another cognitive deficiency or some sort of resource offloading in order to balance the closed-loop of energy conservation in the brain (Brem et al., 2014). This theory could in part explain potential tradeoffs seen with tDCS enhancement that may occur in one region or part of the brain, or possibly through the methods of offloading an entire other executive function as postulated by Luber (2014). Consideration of these effects is especially pertinent for active-duty military populations, whereas potential trade-offs may not be desirable within the context of a certain mission (e.g., target, no-target), though situations may arise where these trade-offs would not be detrimental to mission success. Other currently used interventions in the military, such as caffeine, also have trade-offs. For example, doses up to 400 mg is generally not associated with side effects, but large portions of active-duty personnel report regularly exceeding this amount, which puts individuals at risks for things such as full-body tremors and insomnia, and in severe cases *caffeine toxicity* (Robert et al., 2015; Knapik et al., 2016). Thus, the study reported here was an initial examination of tDCS for possible use within the military with special consideration given to the potential for performance tradeoffs.

The use of tDCS has been routinely documented as being relatively safe, with few side effects reported after use (Eryilmaz et al., 2014; Matsumoto and Ugawa, 2017). Application of tDCS modifies neuronal activity both through active and inhibitory actions by using small electrical currents that target areas of the cortical surface below the scalp (Thair et al., 2017). Newer theories suggest that in addition to stimulation of the cortex, co-stimulation of cranial and cervical nerves may also be simultaneously occurring, having further neuromodulator effects that may influence cognition (Madji et al., 2023). Where to position the electrodes on the scalp to target various underlying brain regions, and which brain regions to target for a given cognitive process, remains inconsistent in the literature (Feltman et al., 2020). For example, much of the research examining the use of tDCS for enhancing working memory in healthy populations tends to focus on stimulation applied to the prefrontal cortex, although this varies whether targeting the left or right side (for a recent review, please see Senkowski et al., 2022). Alternatively, researchers examining various aspects of attention have targeted a range of brain regions, such as the parietal cortex, frontal cortex, and occipital cortex (for a recent review, please see Reteig et al., 2017). Noteworthy, however, is that many of these choices were driven by the *type* of attentional process being targeted (such as sustained attention vs. a visual search task). However, this does not negate the fact that the methods used to study the impact of tDCS on various cognitive processes is murky. Indeed, besides where and how long to apply tDCS, whether it is applied prior to a task (offline) or during a task (online), as well as what stimulation intensity is used can impact outcomes (Feltman et al., 2020). Typically, tDCS stimulation duration ranges from 15 to 30 min, using between 1.5 and 2.0 mA current levels (Thair et al., 2017). In a systematic review conducted on the viability of tDCS for performance enhancement, close to half of all studies that showed promise for enhancement from tDCS used a stimulation strength of 2.0 mA, and approximately half applied tDCS for 20 min, demonstrating that these parameters may be sufficient for achieving cognitive enhancement (Feltman et al., 2020). Other aspects of tDCS application to consider include active-anodal vs. active-cathodal stimulation given the documentation of cathodal tDCS inhibiting neuronal response and anodal tDCS increasing neuronal excitability.

Ultimately, the difference in methods used has led to inconsistent results in the literature. For example, findings for working memory are mixed; with some studies reporting improvements on tasks of working memory such as the *n*-back task (Perrotta et al., 2021), while others report no improvements (Luque-Casdo et al., 2020). While many scientists in this field have pointed to the differences in methodology used, recent literature has been highlighting other potential factors. These include individual differences in susceptibility to respond to the application of stimulation or how engaged an individual is with the task at hand (Vergallito et al., 2022). This is a topic of active research in attempts at identifying the ideal stimulation parameters. Moreover, researchers have also begun to systematically assess some of these differences by comparing different current strengths (Agboada et al., 2019; Weller et al., 2020).

When considering the use of tDCS in healthy populations with high stakes occupations, such as the military, it is important to understand all potential side effects associated with its use. Much of the literature to-date has focused on documenting effects related to physical changes, such as skin irritation (Bikson et al., 2009) as well as mood changes (Plazier et al., 2012). However, given that tDCS likely affects more than the just the targeted brain region for the behavior of interest (Das et al., 2016), it is important to consider other behavioral changes that may occur that are less desirable. Examples of undesirable behavioral changes include increased risk taking and impulsivity and decreased cognitive flexibility. For example, Beharelle et al. (2015), found that decision-making outcomes during a reward-learning task were dependent on the type of stimulation subjects received. Specifically, cathodal stimulation resulted in subjects focusing on immediate large rewards, while anodal stimulation resulted in subjects' considering past negative predictions. Within military populations, these types of behavioral effects could be catastrophic. What has been documented to-date regarding these sorts of behavioral effects has typically been within clinical populations (i.e., use of tDCS to reduce impulsivity in individuals with ADHD) or in studies where the task under study was the only targeted aspect of cognition (i.e., evaluating the effects of tDCS on cognitive flexibility) (Chrysikou et al., 2013; Gilmore et al., 2018). In addition, the evaluation of the effects of tDCS on cognitive flexibility found cathodal tDCS improved flexibility (Chrysikou et al., 2013). Thus, it is not known whether active-anodal tDCS potentially decreases flexibility. Additional research has demonstrated evidence of near transfer abilities to other tasks from the original task of interest during tDCS stimulation (Ehrhardt et al., 2021). For tDCS to move beyond the laboratory for use in healthy populations within real-life settings, the various desirable and undesirable effects on different behavioral outcomes needs to be better understood.

The purpose of this study was to evaluate whether cognitive performance is improved with the application of tDCS. Given the inconsistencies in existing literature (Feltman et al., 2020), we chose to use the most cited tDCS parameters for enhancement (left dorsolateral prefrontal cortex, 2 mA) to evaluate whether cognitive processing could be enhanced across a range of tasks targeting specific cognitive functions of interest to military populations. We also chose to compare active-anodal to active-cathodal (and sham), as the effects of current direction remain under debate (Lafon et al., 2017). Aspects of cognitive performance that were investigated included working memory, attention, decision making and executive control. In addition to examining the effect of tDCS on these targeted cognitive functions, potential undesirable side effects, including effect on mood, medically relevant physical changes, behavioral (e.g., impulsivity), and fatigue were also examined. The overarching goal of this study was to better understand how tDCS affects the cognitive performance of healthy, well-rested, active-duty Soldiers, and what potential side effects are present after its application. As such, the study included healthy individuals with no pre-existing cognitive or health conditions, and who were documented as well-rested (based on sleep requirements measured through an Actiwatch^®^, which is a wrist worn device similar to a Fitbit^®^).

Based on past literature, we hypothesized that the active anodal and cathodal conditions would differ from the sham condition. We did not form specific hypotheses regarding the outcomes for each cognitive tasks, nor did we specify the directionality of the differences between conditions. Thus, all hypotheses were two-tailed in nature. We focused on evaluating two objectives: (1) to evaluate whether cognitive performance was improved during the application of either or both active-anodal and -cathodal tDCS to the left DLPFC for 30 min with 2 mA; and (2) to document any secondary side effects, including mood changes, medically relevant side effects, increased risk taking, impulsivity, decreased cognitive flexibility, and increased fatigue. Additionally, gender was evaluated to determine whether gender effects occurred, again with no specific hypotheses tied to these evaluations.

## 2 Materials and methods

The findings presented in the current study were part of a larger study that evaluated whether the application of tDCS subsequently (post-stimulation) impacted military task performance. The protocol for the original study was approved by The U. S. Army Medical Research and Development Command Office of Research Protections Institutional Review Board. All methods and procedures conducted by researchers were in accordance with the ethical guidelines and regulations per the Institutional Review Board policies. Prior to participation, all participants provided informed written consent. The data reported here are a subset of data from a larger study (Feltman et al., 2021). This study's design, hypotheses and analyses were preregistered at ClinicalTrials.gov under record number NCT04155333.

### 2.1 Participants

A total of twenty-seven healthy (14 males), active-duty U.S. Army Soldiers from Fort Novosel (formerly Fort Rucker), Alabama were recruited via word of mouth, the use of flyers, email, and through various social media platforms. Participants ranged in age from 21 to 40 years (*M* = 29.04, *SD* = 5.77). Potential volunteers were informed they would be compensated with a $1,200 check upon completion of the study. Please see Table 1 for additional demographics regarding this sample.

**Table 1:** Demographic descriptive statistics.

**Demographic item**	**Males**		**Females**		**Total**	
**Descriptive statistics reported as** ***M (SD)***						
Age	32.23 (5.70)		26.46 (3.99)		29.35 (1.11)	
Body mass index^*^	28.17 (4.41)		27.65 (4.27)		27.91 (4.26)	
WAIS IV-R IQ^**^	106.62 (6.49)		105.36 (6.71)		105.96 (6.51)	
**Frequencies reported as** ***n*** **(%)**						
Nicotine use	**Yes**	**No**	**Yes**	**No**	**Yes**	**No**
	2 (15.4%)	11 (84.7%)	0	14 (100%)	2 (7.4%)	25 (92.6%)
Handedness	**Left**	**Right**	**Left**	**Right**	**Left**	**Right**
	1 (7.7%)	12 (92.3%)	3 (21.4%)	11 (78.6%)	4 (14.8%)	23 (85.2%)

^*^Calculated using BMI calculator provided by the National Heart, Lung, and Blood Institute [Calculate Your BMI—Standard BMI Calculator (nih.gov)]. Also, note missing one female response for BMI. Average BMIs are considered overweight.

^**^Estimated through the Shipley Institute of Living Scale.

All participants met the following inclusion criteria: no current medical conditions or use of medications that affect cognitive function or attention; no history of psychological/psychiatric disorder; no history of neurological disorders; no history of head injury resulted in loss of consciousness; no metal located in the head or medical implant (shrapnel, cardiac pacemaker); no skin conditions affecting the scalp; no current use of hormone medicine (excluding birth control). Participants were also screened to ensure they did not have the possibility for caffeine withdrawal symptoms that may impact cognitive functioning. These exclusion criteria were assessed from self-report measures and verified by an on-site physician on the first lab visit.

### 2.2 Measures - targeted

#### 2.2.1 Cognitive tasks

The following cognitive tasks aimed to assess working memory, attention, impulsivity, risk taking, motor control, and executive functioning. Each were administered electronically either via an open-source software package (*PsychoPy*; Peirce et al., 2019) or through downloaded programs.

**Stroop Task** (Macleod, 1991) is designed to measure *selective attention* by presenting mismatched colored words with the corresponding color written as the word. For example, the word “RED” would be written in purple. The task includes 10 trials of congruent and incongruent pairs of color-words that takes ~3 min to complete. The key outcome measure in the present study was the Stroop Effect, measured in milliseconds (ms). See Supplementary Item 1 for an example of the presentation of the Stroop Task.

**Digit Span Task** (Miller, 1956) measures *working memory* by presenting a string of numbers that increase in length for each trial that require a participant to correctly recall. The time to complete this task was 2 min. The outcome measure for this study is span size recalled. See Supplementary Item 2 for an example of the presentation of the Digit Span Task.

**Rapid Visual Information Processing Task** (Bakan, 1959) presents participants with a sequence of digits that are is designed to measure *sustained attention*. Participants must accurately detect even-odd-even sequences that were presented in a fast and a slow mode. Participants completed 6-blocks of trails and took ~7 min to complete. Outcome measures for this task included reaction time in ms and *d*′, a measure of sensitivity. See Supplementary Item 3 or an example of the presentation of the Rapid Visual Information Processing Task.

**Shifting Attention Task** (Royer, 1971) is a 2-min task that measures *executive function*, set shifting, and attention. Participants are presented with a key and must determine if a set of digits and symbols correctly corresponds to the key (a total of 98 digits to code). The outcome measures for this task were reaction time in ms and accuracy.

### 2.3 Measures—Side effects cognitive tasks

The following cognitive tasks aimed to assess potential side effects associated with the application of tDCS. These measures included impulsivity, motor control, risk taking, and cognitive flexibility.

**Stop Signal Task** (Logan et al., 1997) instructs participants to respond as quickly as possible to symbols that are identified as either “go” or “stop” signals. This task takes ~3 min to complete and includes 512 trials. It is designed to measure *impulsivity* and *motor control*. The outcome measures included reaction time in ms and accuracy. See Supplementary Item 4 for an example of the presentation of the Stop Signal Task.

**Delay Discounting Task** (Koffarnus and Bickel, 2014) measures participants' *risk taking* and *impulsivity* by giving two scenarios in which one results in receiving and immediate amount of hypothetical money while the other requires a longer time period but a larger sum. The key outcome measure was discount rate (*k*). The discounting task includes five trials and takes ~1 min to complete. See Supplementary Item 5 for an example of the presentation of the Delay Discounting Task.

**Uses Task** (Chrysikou and Thompson-Schill, 2011; Chrysikou et al., 2013) measures *flexible thinking* by presenting the participant with black-and-white pictures of everyday objects. The participants state an uncommon use for the object depicted. Participants were presented the object for 9 s, with a 3 s interval before the next object presentation. Verbal responses were recorded to evaluate novelty and plausibility of the response. Four sets of pictures were created to be used in repeated testing which took approximately 5 min to complete. Order of the sets were counterbalanced amongst participants. Primary outcome measures included the novelty and plausibility of responses, which were assessed by two raters using a 5pt Likert-like scale.

Interrater agreements were checked using the crosstabs function in SPSS v25 using the Kappa statistic to determine consistency among raters (Landis and Koch, 1977). For novelty ratings, the interrater agreement was Kappa = 0.03, *p* = 0.003, suggesting “slight agreement.” For plausibility, Kappa = 0.02, *p* = 0.005, also suggesting “slight agreement.” Prior to analyses, the ratings from the two reviewers for each novelty and plausibility were combined to compute a single variable, which was the mean rating, for each participant. See Supplementary Item 6 for an example of an object presented in the task.

### 2.4 Measures—Questionnaires

Non-copyrighted materials used are supplied in the Supplementary material.

**Shipley Institute of Living Scale (Zachary**, **1986****)** was designed to assess general intellectual functioning in adults and adolescents, and to aid in detecting cognitive impairment in individuals with normal original intelligence. The SILS yields three major summary scores: Vocabulary, Abstraction Quotient, and combined Total scores. The Abstraction subscale includes 20 series completion items of inductive reasoning that tap fluid ability. Convergent validity of both the Vocabulary and Abstraction measures with crystallized and fluid intelligence (respectively) has been assessed and confirmed in a general population (Matthews et al., 2011). The Abstraction Quotient score, which compares the ratio of crystallized intelligence with fluid intelligence, was primary measure of interest from the SILS in the present study. Completion of this assessment takes 15 min.

**Karolinska Sleepiness Scale** (Kaida et al., 2006) is a single-item, self-report questionnaire that assesses individual's current feelings of fatigue. Participants rate sleepiness level during the past 5 min on a scale of 1 (extremely alert) to 9 (extremely sleepy—fighting sleep). The outcome measure was a single sleepiness rating.

**Profile of Mood States—Short Form** (McNair et al., 1971) is a shortened version of the original Profile of Mood States questionnaire that measures psychological distress and mood. The short form has a total of 35 items with a 5-point Likert scale formatting. Each item provides an adjective that a respondent must rate the degree to which is describes them. Seven outcome measures resulted: (1) tension/anxiety; (2) anger/hostility; (3) vigor/activity; (4) fatigue/inertia; (5) depression/dejection; (6) confusion/bewilderment; and (7) total mood disturbance.

**Symptom Checklist (Thair et al.**, **2017****)**. This study used a modified version while only included the physical side effects that are part of the Symptom Checklist questionnaire. Participants rated the presence and severity of each symptom they experienced before and after tDCS application. See Supplementary Item 7 for a copy of the checklist.

**Demographics and Health Screening Questionnaire**. The demographics and health screening questionnaire was developed in-house by the study physicians. The study physician used the questionnaire to aid in determining eligibility for the study and current health status. See Supplementary Item 8 for a copy of the questionnaire.

**Post-Stimulation Questionnaire**. The post-stimulation questionnaire was developed in-house following a similar procedure by Wallace et al. (2016) to query whether participants thought they received active or sham stimulation during the stimulation period. An additional three questions were asked regarding various perceptions of the use of stimulation. Only data regarding the blinding efficacy are reported here. See Supplementary Item 9 for a copy of the questionnaire.

### 2.5 Devices

#### 2.5.1 Transcranial direct current stimulator

The HDCStim device (Newronika s.r.l.) was used for tDCS to modulate neural activity in the left dorsolateral prefrontal cortex (LDLPFC). The device is a Class IIa medical device certified by the Notified Body n.0068 of the European Community. The device conforms to the regulations set forth in the Council Directive 93/42/EEC for medical devices. It conforms to their standards and directives for: general requirements for safety and safety requirements for medical electrical systems (CEI-EN 60601-1), requirements for basic safety and essential performance (CEI-EN 60601-1-2), and programmable medical systems (CEI-EN 60601-1-4). This information can be referenced at: https://ec.europa.eu/growth/single-market/european-standards/harmonise-d-standards/medical-devices_en. The HDCStim device is not approved by the FDA for use in the United States for any indication, therefore, all uses of this device under a research protocol in the United States are considered investigational uses and are subject to the U.S. regulations under 21 CFR 812. The HDCStim device qA labeled with the following statement: “CAUTION-Investigational Device.”

#### 2.5.2 Actiwatch^®^

Actiwatch^®^ is a small limb-worn, wireless device that uses an accelerometer to monitor occurrence and degree of motion of the wearer. The watch was worn for the entire duration an individual was involved in the study and would beep if it was removed. In this study, sleep efficiency was determined by the amount of time that the wearer presumably was “in bed” attempting to sleep. This device was only used to confirm sleep requirements. No data is presented.

### 2.6 Procedure

There were a total of five laboratory visits spaced out by a minimum of 24-h. Three of these visits included actual (or sham) tDCS stimulation. Data collection began prior to and was paused during the onset of the COVID-19 pandemic (March 2020). Data collection resumed in October of 2020, with added safety precautions and procedures to ensure the wellbeing of participants and members of the research team. Prior to beginning each session, participants were screened regarding the following guidelines:

1. Obtained a minimum of 6 h of sleep prior to collect which was confirmed via the use of the Actiwatch^^®^^.
2. Refrained from consuming caffeine within 16 h of the study, nicotine within 2 h, and alcohol 24 h.

A total of four participants had to be rescheduled due to failure to meet the above requirements. During Visit 1, participants completed informed consent, filled out a medical history questionnaire, and met with the study physician. The study physician reviewed the questionnaire with them to ensure accurateness and to determine whether nicotine (three participants reported regular nicotine use) or caffeine withdrawal symptoms may be of concern. Participants then completed a 5-min familiarization stimulation with the tDCS device and received their Actiwatch^®^. Participants were scheduled for the same time of day (morning or afternoon) for all study activities.

During visit 2, participants were screened to ensure they followed the previously outlined study guidelines. Each participant then completed the following questionnaires: the KSS and POMS-SF. Participants then completed various military performance tasks that are not included in the results of this study but are reported in a technical report (Feltman et al., 2021). Participants finally completed the following cognitive tasks for the purposes of familiarizing with the tasks: Stroop Task, Dual *n*-back Task, Digit Span Task, Rapid Visual Information Processing Task, Shifting Attention Task, Digit Symbol Substitution Task, Stop Signal Task, Delay Discounting Task and Uses Task.

The remaining sessions where tDCS was applied were identical across visits (3, 4, and 5), except for one additional questionnaire (the post-stimulation questionnaire) and return of the Actiwatch^®^ during Visit 5. Upon arrival to each visit, adherence to the study guidelines were verified through self-report (abstaining from substances) and via the Actiwatch^®^. Participants then completed the following questionnaires: the symptom checklist, KSS, and POMS-SF to establish pre-stimulation values.

**Figure 1:**
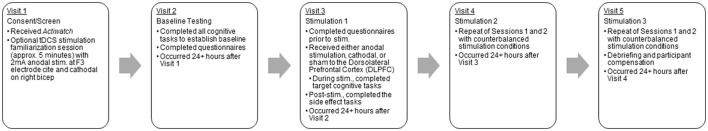
Data collection pipeline.

Individuals then received 2 mA of active-anodal, active-cathodal, or sham stimulation (stimulation procedures are detailed below). The order of these were randomized using researchrandomizer.org. Both participant and researcher were blind to each condition. After 5 min of stimulation elapsed, participants completed the following cognitive tasks: Stroop Task, Digit Span, RVIP, and Shifting Attention (note, an additional task, Dual *n*-back was also completed during this time frame; data are not reported as the task was determined too difficult, with many participants failing to move beyond the practice trials). The tasks typically ended at the same time stimulation completed (30 min total stimulation), but for some participants stimulation ended slightly earlier than the task. To account for this, the order of tasks was randomized for each testing session. After the completion of the tasks and stimulation, the device was removed and participants completed the symptom checklist, KSS, and POMS-SF a second time to measure potential changes in mood or side effects after stimulation. Individuals then went on to complete military performance tasks. Finally, the Stop Signal Task, Uses Task and Delay Discounting tasks were completed approximately an hour post-stimulation. The order of these tasks was also randomized. Participants were then asked to distinguish between the active and sham stimulation and whether it impacted their performance. Participants remained at the laboratory for an additional hour, engaging in recreational activities. The study physician screened the participant to ensure no lingering side effects before clearing the participant to leave the laboratory. A visual representation of the data collection is depicted in Figure 1.

### 2.7 Stimulation procedures

**Figure 2:**
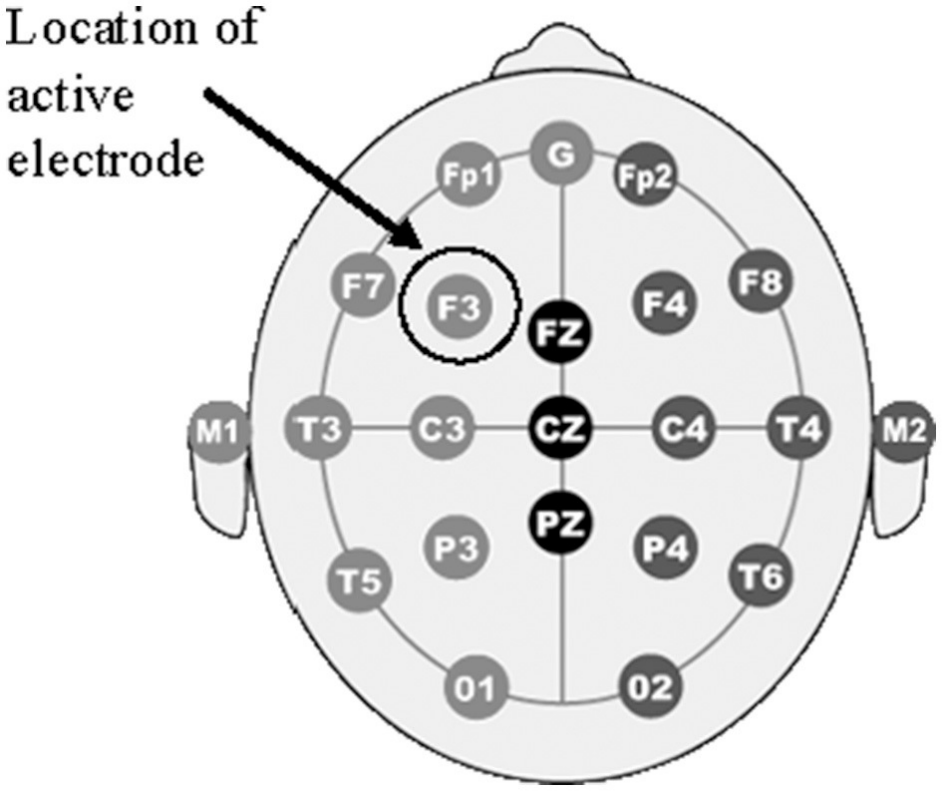
Electrode placement. The figure shows a diagram of electrode placements in the 10-20 international system arrangements. The location of the “F3” electrode is circled with an arrow denoting “Location if active electrode” which corresponds to the DLPFC.

The HDCStim^®^ device was used to administer 2 mA of direct current to the left DLFPC (F3, shown in Figure 2). Rubber electrodes were placed inside each sponge that had been soaked in saline solution for ~10 min. Per the manufacturer's recommendation, a conductive gel was then applied to the outside of each saline-soaked sponge that made contact with the participant's skin. The smaller (5.0 × 5.0 cm) electrode was applied to F3 using the Beam F3 application. This application uses the distance from tragus to tragus, nasion to inion, and head circumference to determine the precise location of F3. The larger electrode (8.5 × 6.0 cm) was applied to the right bicep. The electrode on the head was held in place using a rubber strap in the center and 3m™ Coban™ wrap on the top and bottom to ensure proper contact. The electrode placed on the right bicep was held in place using two rubber straps at the top and bottom of the electrode. Each participant experienced active-anodal, active-cathodal and sham stimulation. Both the participant and the research staff were blind to the condition. Impedances were checked every minute by a member of the research team who was not engaged in data collection. If the impedances went above 10 kOhm, they were to report it to the research staff who would apply additional gel/saline. This never occurred over the duration of the study and adequate impedance values were observed. Active stimulation was applied for a total of 30 min, with a 30 s ramp-up and ramp-down time. The sham stimulation consisted of a 90 s period that included the 30 s ramp-up/down times and 30 s of active stimulation. Stimulation always began 5 min prior to beginning the cognitive tasks.

Double-blinding was achieved by first using a web-based random order generator (researchrandomizer.org) to create random orders of conditions for each participant. This was completed by the PI. From this, a table was produced with conditions labeled as A, B, and C to indicate the orders of sham, active-anodal, and active-cathodal stimulation. Next, an additional random order was generated for sham condition configurations, as two configurations were used. Independently from the creation of these tables (A, B, C and 1, 2), a second investigator created a document to identify the labels [sham, active-anodal, and active-cathodal stimulation, and sham configurations (anodal or cathodal)]. The investigators did not share these lists with one another and rather kept them stored in secured, access-limited folders. Then to maintain blinding, there was a separate individual on the research team whose sole responsibility was programming the conditions within the device. Prior to stimulation, the “device programmer” accessed the two randomized lists and programmed the device for the participant.

## 3 Results

Prior to conducting analyses, all hand-entered data were verified for accuracy using a 10% sample validation check, where 10% of the data were double-checked, and any erroneous entries were re-entered using double entry. All electronically recorded data were inspected for unrealistic values. Next, data were evaluated to ensure they met the assumptions for parametric testing and examined for outliers. Outliers, identified as exceeding three standard deviations from the mean, were removed and are referenced in the text below as applicable.

To determine the effects of the stimulation on cognitive performance, outcome measures for each task and questionnaire were evaluated using 2 (gender: male, female) × 3 (condition: sham, active-anodal, active-cathodal) repeated measures analysis of covariance (ANCOVA). Gender was included as a variable given that literature has historically shown mixed effects of tDCS between genders with associated cognitive outcomes (Upadhayay and Guragain, 2014). Two covariates were used, age and abstract reasoning score, in order to control for age-related cognitive decline (Salthouse, 2009), and intelligence scores. For tasks that had multiple independent outcomes, repeated measures multivariate analyses of covariance (MANCOVAs) were conducted with subsequent paired comparisons using paired-samples *t*-tests (Armstrong, 2014). The Benjamini-Hochber (B-H) procedure was applied to control for a false discovery rate of 25%, given the use of multiple comparisons (Benjamini and Hochberg, 1995). Frequency values are reported for blinding efficacy.

### 3.1 Addressing objective one—Performance on targeted cognitive tasks

To address Objective One, performance on the targeted cognitive tasks that were performed during stimulation were evaluated. All means and standard deviations for the cognitive tasks are reported in Table 2. Significance criteria was set at ɑ = 0.05.

**Table 2:** Targeted cognitive tasks descriptive statistics.

**Task**	**Outcome measure**	**Sham** ***M (SD)***		**Active-anodal** ***M (SD)***		**Active-cathodal** ***M (SD)***	
		**Male**	**Female**	**Male**	**Female**	**Male**	**Female**
Stroop Task *n* = 23 (11 males)	Stroop effect	155.11 (74.25)	179.72 (105.69)	172.22 (151.02)	157.92 (128.49)	140.42 (104.20)	149.48 (108.70)
Digit Span Task *n* = 26 (12 males)	Span length	6.42 (0.70)	6.29 (0.91)	6.33 (0.89)	6.57 (0.76)	5.50 (1.31)	6.21 (1.19)
Rapid Visual Information Processing *n* = 26 (12 males)	*Fast* reaction time (ms)	484.23 (43.43)	510.67 (52.99)	472.64 (43.20)	519.51 (65.86)	486.69 (41.70)	506.61 (58.54)
	*Fast* d'	3.43 (1.20)	3.49 (0.85)	3.44 (1.42)	3.03 (0.76)	3.23 (1.04)	3.76 (0.83)
Shifting Attention Task *n* = 25 (13 males)	Reaction time (ms)	1,238.33 (123.06)	1,216.73 (229.81)	1,186.56 (214.49)	1,199.01 (257.95)	1,238.63 (171.77)	1,166.25 (211.62)
	Accuracy	93.38 (9.69)	97.17 (18.78)	99.31 (14.48)	100.50 (21.77)	95.15 (12.10)	102.08 (16.86)

#### 3.1.1 Selective attention measured by Stroop Task

A total of four outliers were removed from the analyses, resulting in a sample size of *n* = 23. There was no significant interaction between condition and performance on the Stroop effect for the ANCOVA, *F*_(2, 38)_ = 0.12, *p* = 0.88.

#### 3.1.2 Working memory measured by Digit Span Task

**Figure 3:**
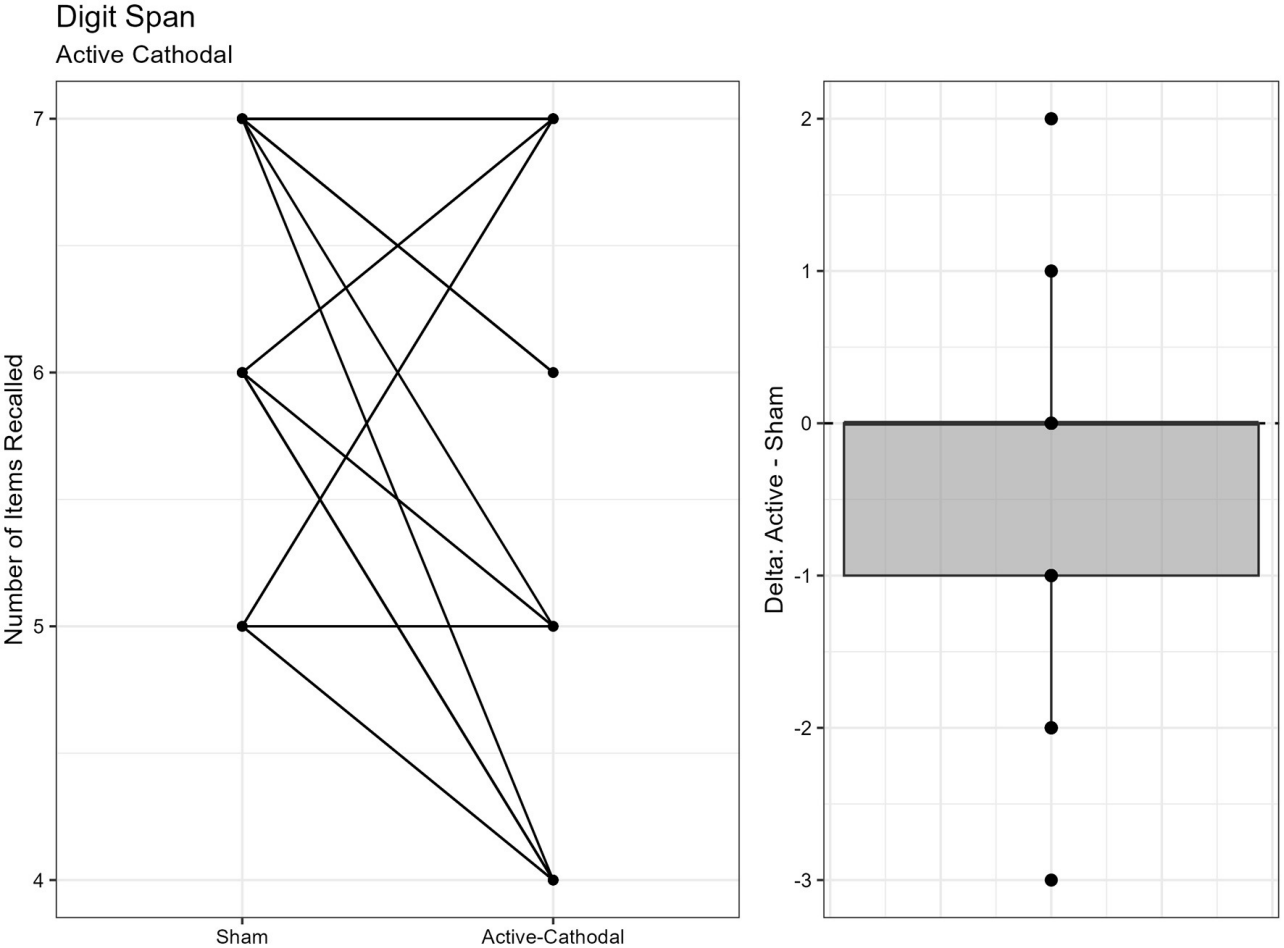
Difference between Cathodal and Sham conditions during the DigitSpan task.

**Figure 4:**
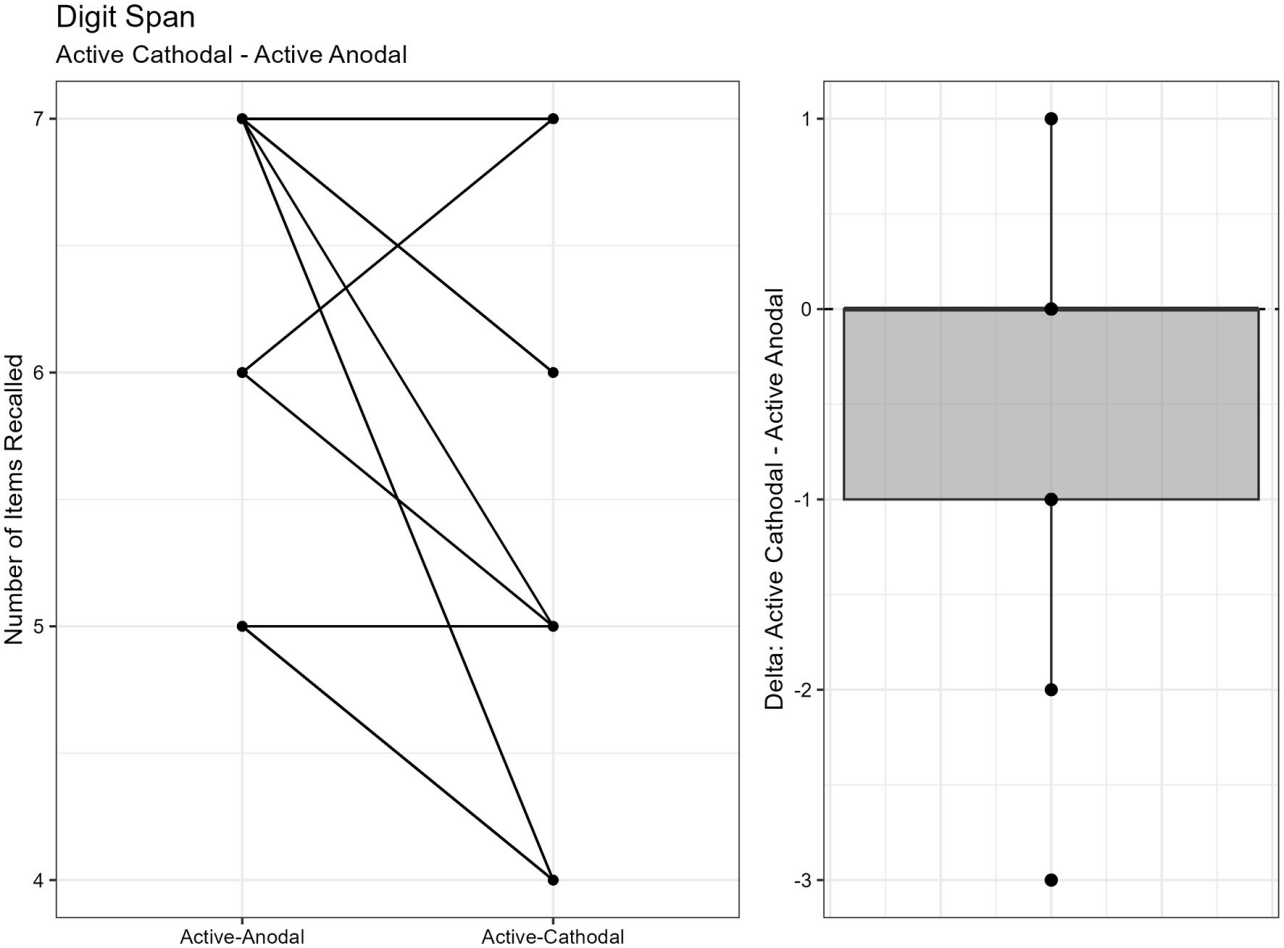
Difference between Anodal and Sham conditions during the DigitSpan task.

There was one outlier removed, resulting in a sample size of *n* = 26. ANCOVA results showed a significant effect of condition on digit span length, *F*_(2, 48)_ = 5.35, *p* = 0.008, $\eta_{p}^{2}$ = 0.18. Pairwise comparisons showed that active-cathodal stimulation yielded a significantly lower digit span length compared to the sham condition (*p* = 0.039, *q* = 0.10) and the active-anodal condition (*p* = 0.005, *q* = 0.02) (Figures 3, 4).

#### 3.1.3 Sustained attention measured by Rapid Visual Information Processing Task

**Figure 5:**
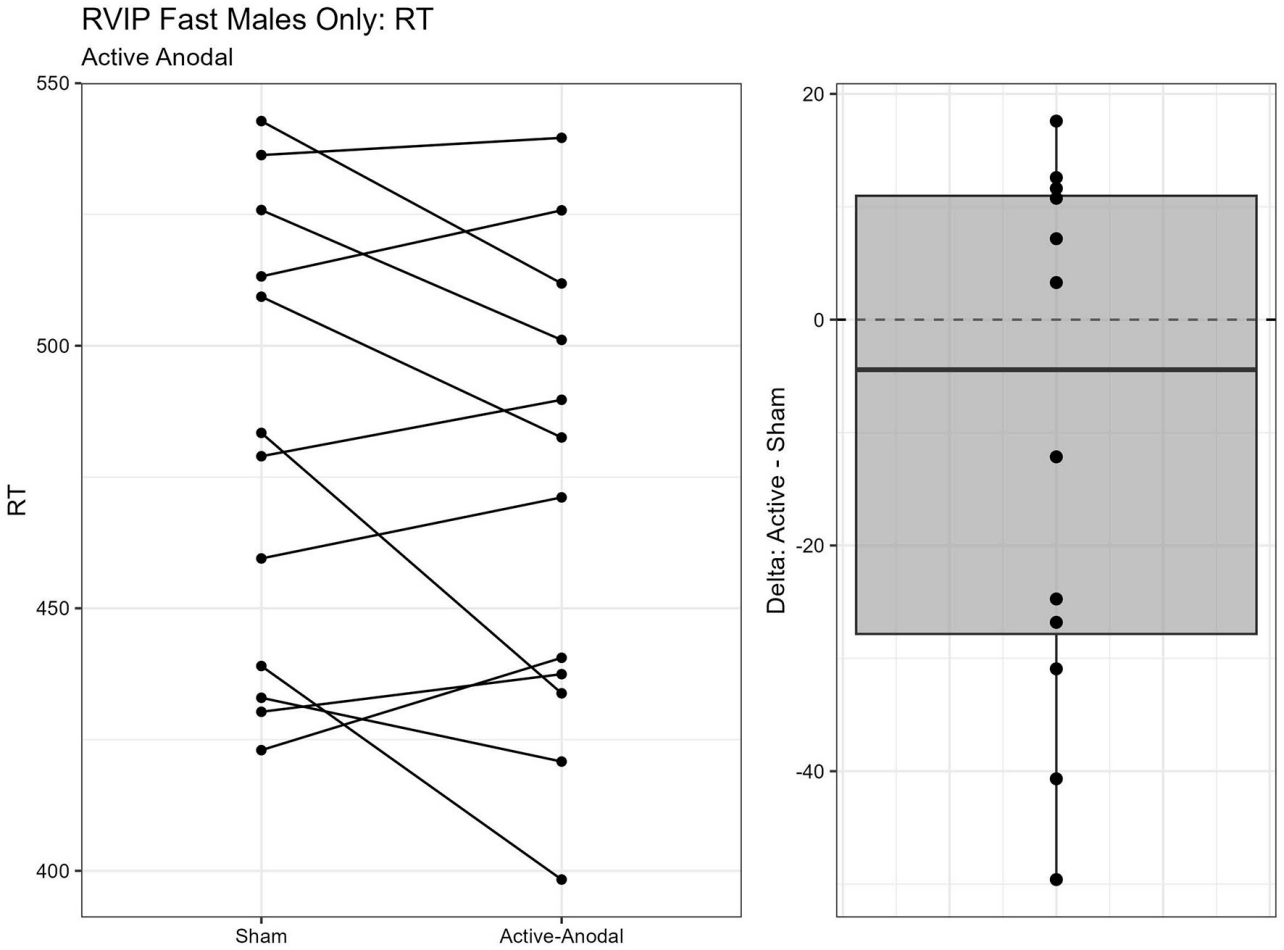
Difference between Anodal and Sham conditions for reaction time during the RVIP task in males only.

**Figure 6:**
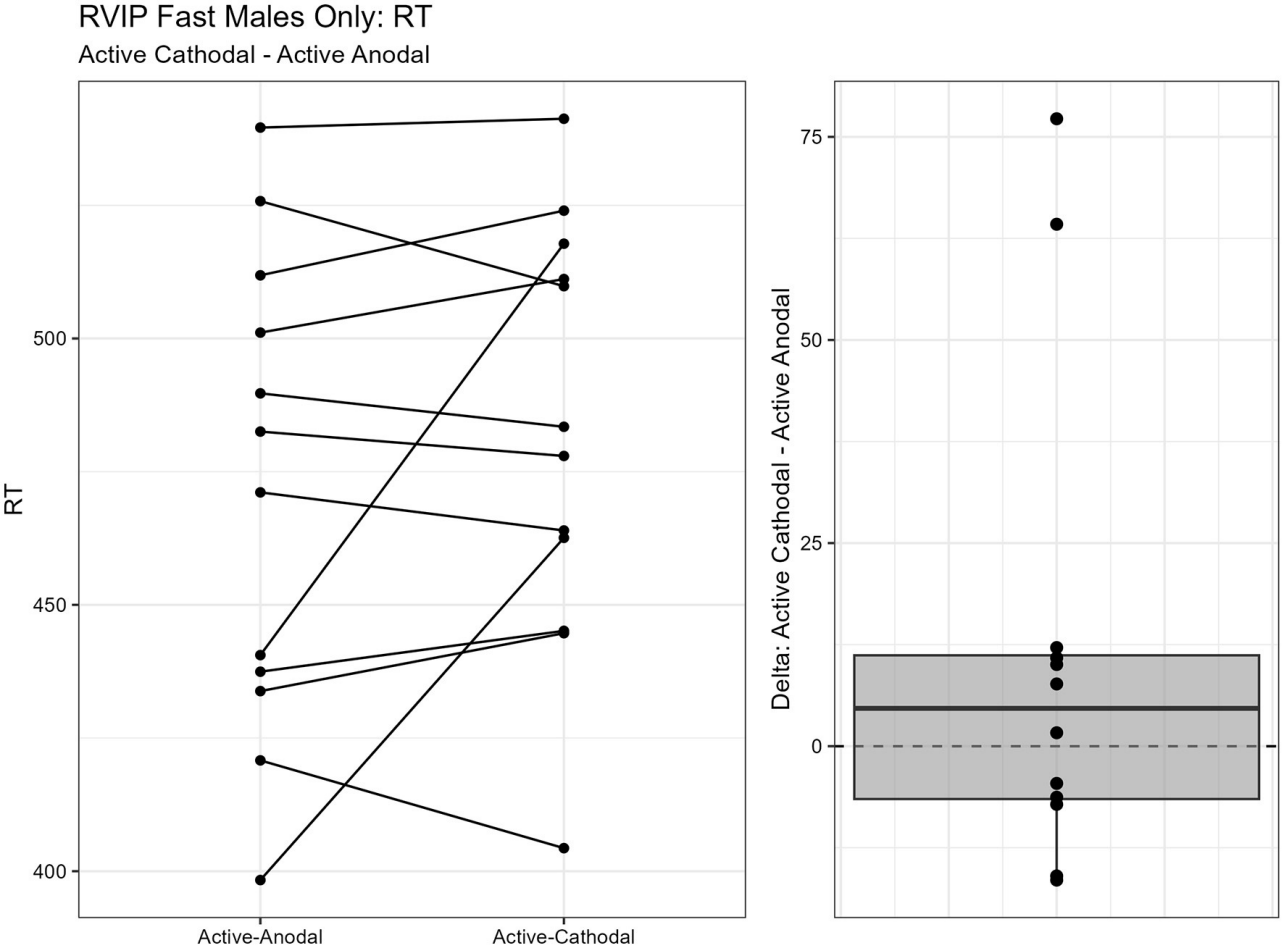
Difference between Cathodal and Anodal conditions for reaction time during the RVIP task in males only.

**Figure 7:**
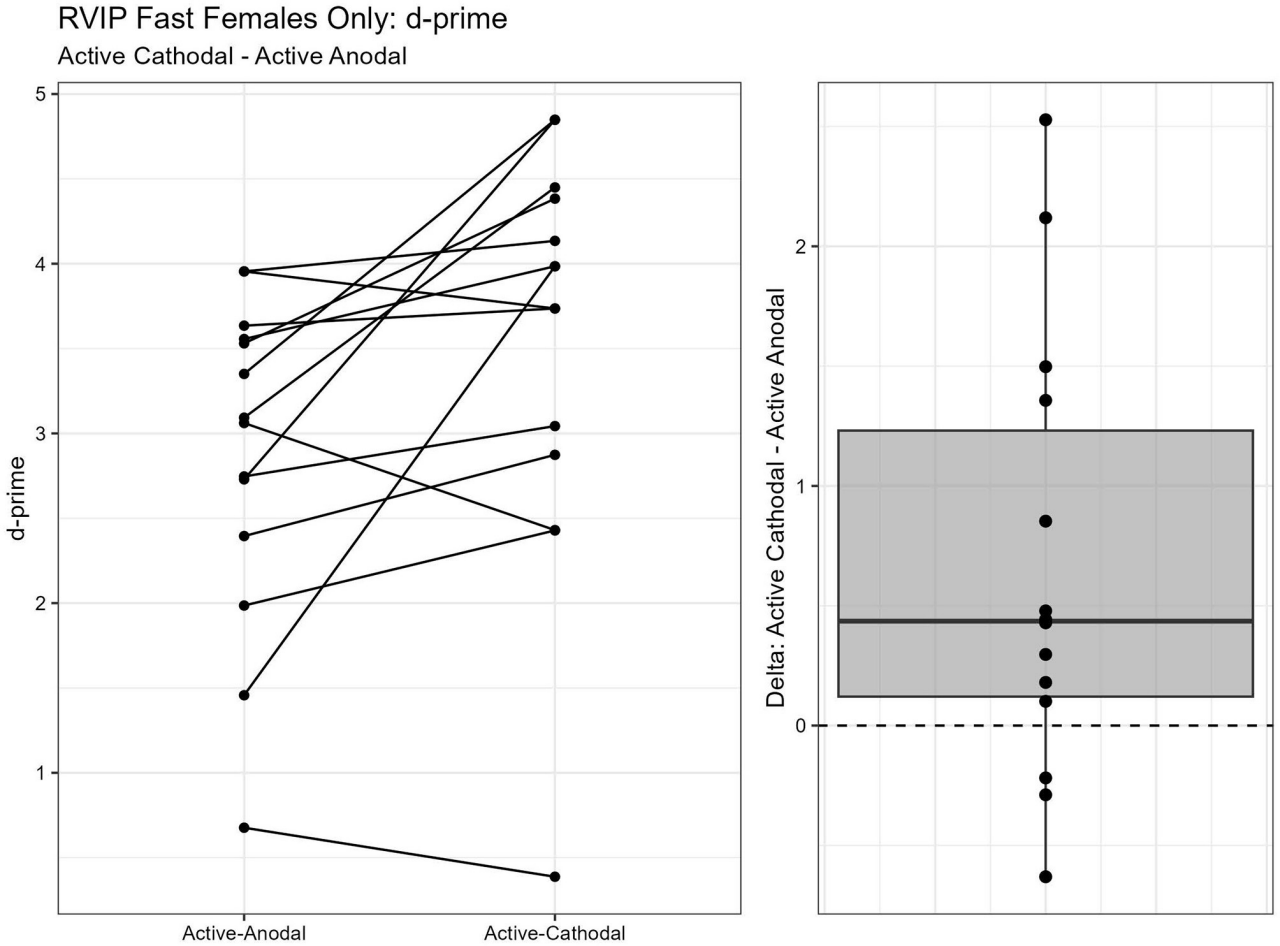
Difference between Cathodal and Anodal conditions for d' during the RVIP task in females only.

**Figure 8:**
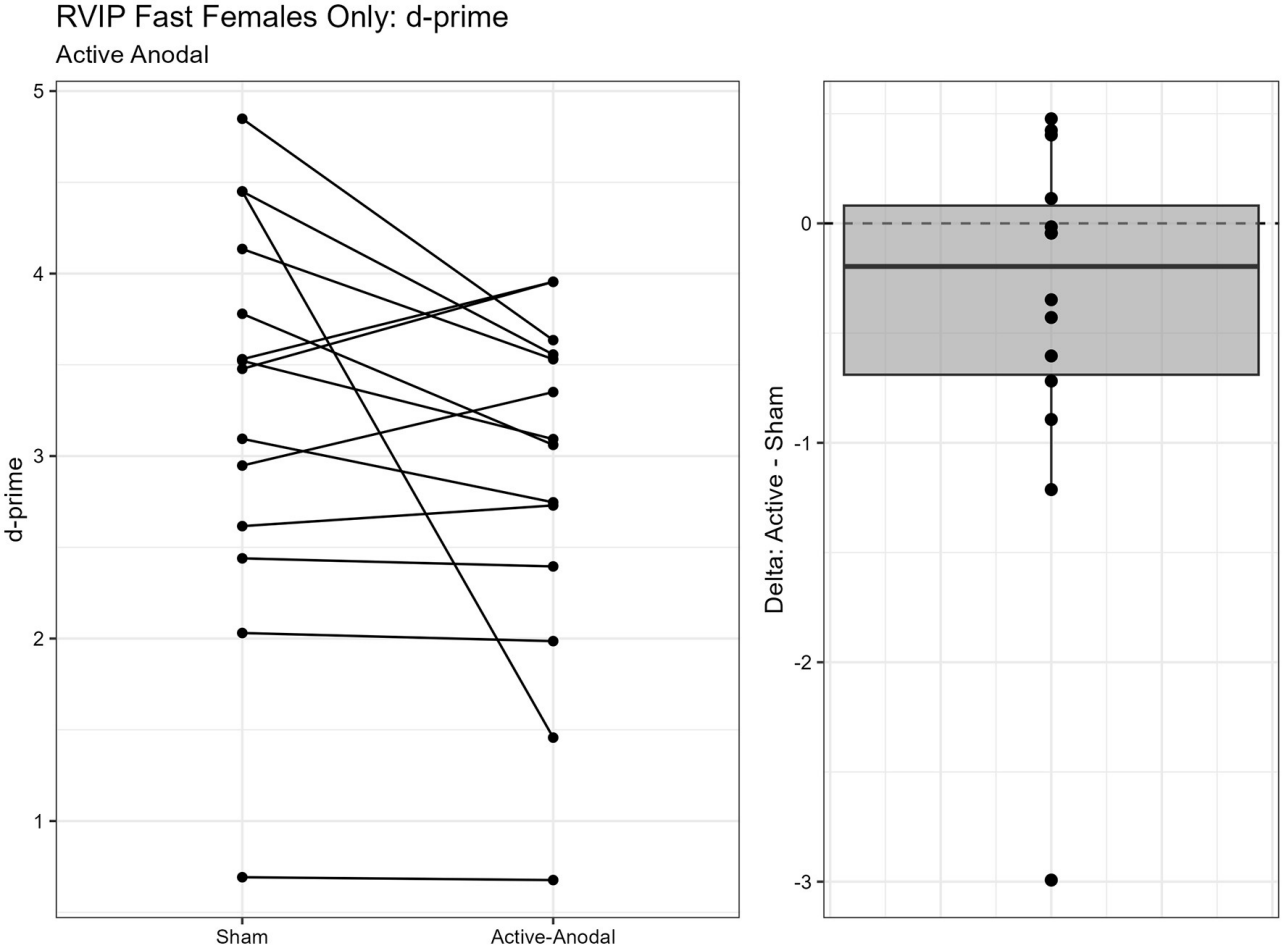
Difference between Anodal and Sham conditions for d' during the RVIP task in females only.

One outlier was removed resulting in a sample size of *n* = 26. Two separate models were run for the two stimuli presentation speeds (fast vs. slow). MANCOVAs were conducted using reaction time and *d*′ (sensitivity index) as outcome measures. The fast presentation mode MANCOVA demonstrated a significant main effect for reaction time and gender, *F*_(2, 44)_ = 4.09, *p* = 0.024, $\eta_{p}^{2}$ = 0.16. Pairwise comparisons showed that reaction time was significantly faster in the active-anodal condition than in the sham (*p* = 0.008, *q* = 0.03) and compared to the active-cathodal condition (*p* = 0.01, *q* = 0.05) for males, *F*_(2, 44)_ = 4.81, *p* = 0.024, $\eta_{p}^{2}$ = 0.16 (Figures 5, 6). Additionally, there was a significant interaction effect for *d*′ and females, *F*_(2, 44)_ = 4.78, *p* = 0.013, $\eta_{p}^{2}$ = 0.18, *q* = 0.06. Pairwise comparisons showed that *d*′ was greater in the active-cathodal condition than in the active-anodal condition for females (*p* = 0.014). There were no significant effects for the slow condition, *F*_(4, 76)_ = 0.44, *p* = 0.78 (Figures 7, 8).

#### 3.1.4 Executive function measured by Shifting Attention Task

**Figure 9:**
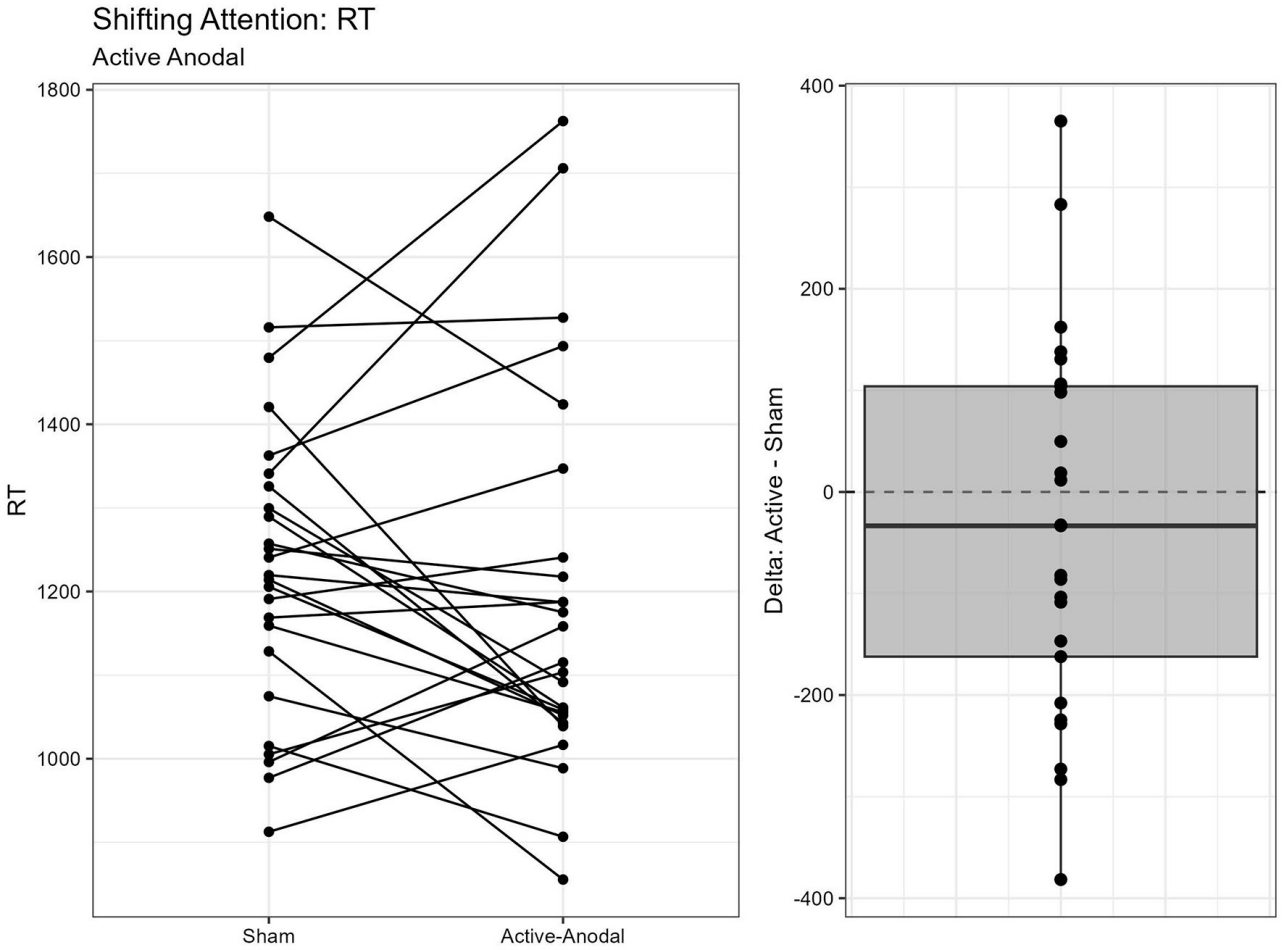
Difference between Anodal and Sham conditions for reaction time during the Shifting Attention task.

**Figure 10:**
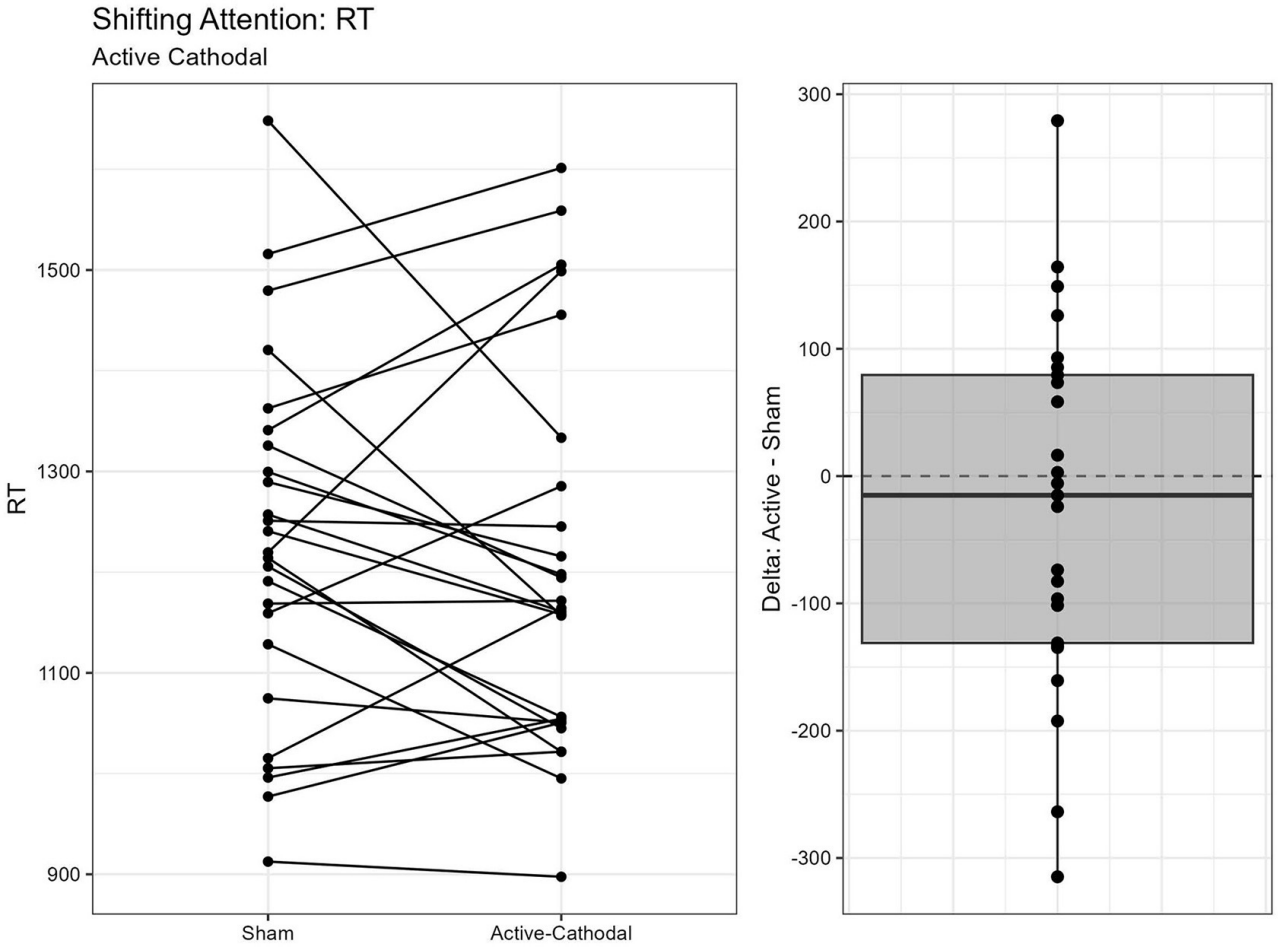
Difference between Cathodal and Sham conditions for reaction time during the Shifting Attention task.

A total of two outliers were removed, *n* = 25. A MANCOVA was conducted for reaction time and accuracy. There was a significant main effect of condition for both reaction time, *F*_(2, 42)_ = 3.68, *p* = 0.034, $\eta_{p}^{2}$ = 0.15, and accuracy, *F*_(2, 42)_ = 3.85, *p* = 0.029, $\eta_{p}^{2}$ = 0.16. Pairwise comparisons showed that reaction times were faster with both the active-anodal (*p* = 0.049, *q* = 0.11) and active-cathodal (*p* = 0.036, *q* = 0.09) conditions compared to the sham condition. Accuracy was also greater in both the active-anodal (*p* = 0.027, *q* = 0.08) and active-cathodal (*p* = 0.049, *q* = 0.12) conditions compared to sham (Figures 9, 10).

### 3.2 Addressing objective two—Assessing side effects

To address Objective Two, performance on the side effects cognitive tasks performed post-stimulation were evaluated. All means and standard deviations for the cognitive tasks are reported in Table 3.

**Table 3:** Side effects cognitive tasks descriptives statistics.

**Task**	**Outcome measure**	**Sham** ***M (SD)***		**Active-anodal** ***M (SD)***		**Active-cathodal** ***M (SD)***	
		**Male**	**Female**	**Male**	**Female**	**Male**	**Female**
Delay Discounting *n* = 22 (11 males)	*k* value	0.016 (0.018)	0.025 (0.028)	0.084 (0.243)	0.022 (0.024)	0.019 (0.019)	0.017 (0.024)
Stop Signal Task *n* = 23 (12 males)	Accuracy	83.29 (4.95)	86.83 (5.21)	85.40 (5.77)	85.13 (6.08)	85.25 (4.30)	85.95 (4.51)
Uses Task *n* = 22 (13 males)	Novelty ratings	0.11 (0.64)	−0.13 (0.50)	0.06 (0.54)	−0.16 (0.50)	0.08 (0.49)	−0.15 (0.42)
	Plausibility ratings	−0.38 (0.93)	0.02 (0.40)	−0.22 (0.90)	0.04 (0.35)	−0.29 (0.93)	−0.05 (0.47)

#### 3.2.1 Risk taking measured by Delay Discounting Task

**Figure 11:**
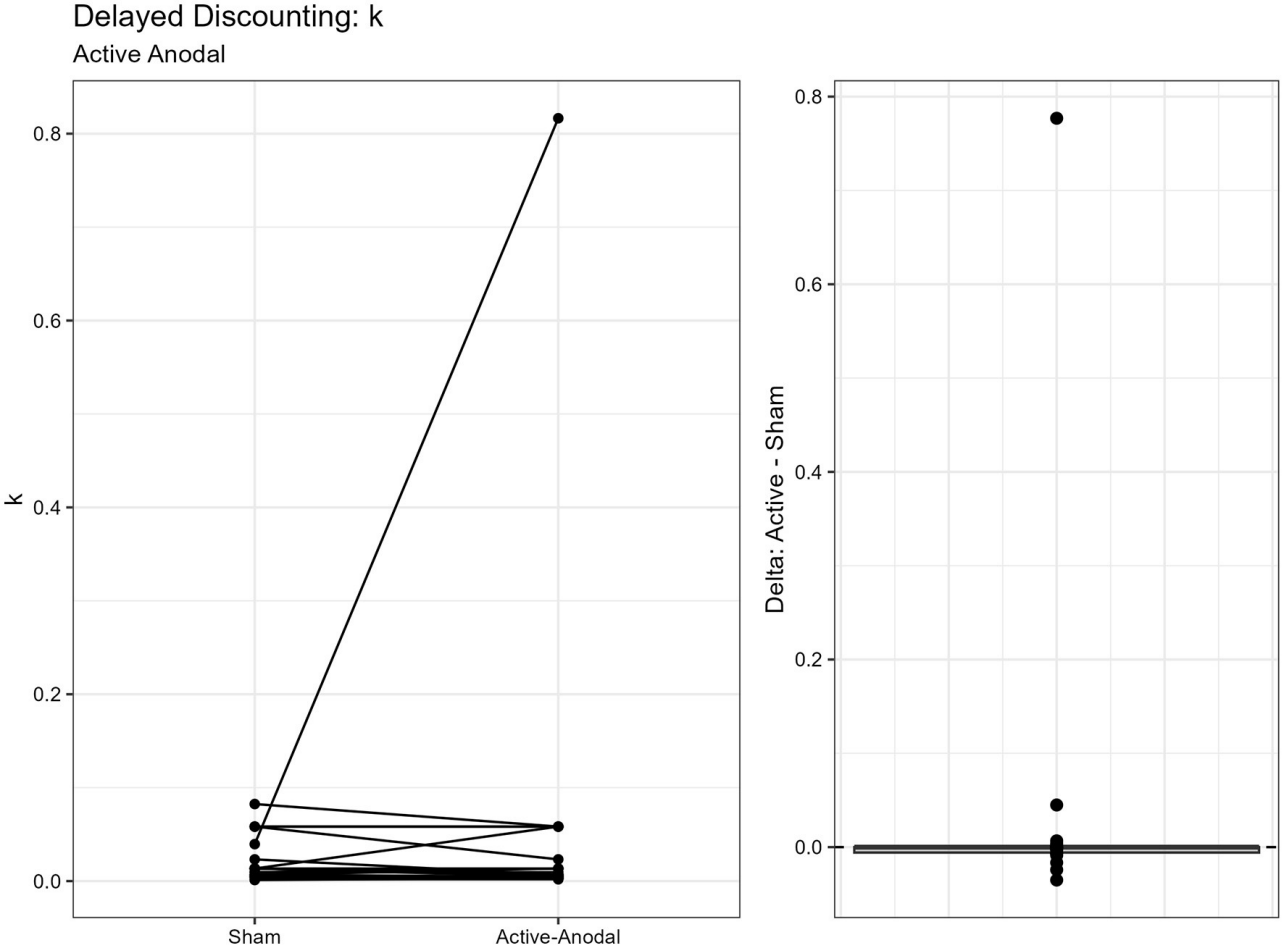
Difference between Anodal and Sham conditions for k during the Delayed Discounting task.

Five outliers were removed prior to running the ANCOVA on the primary outcome of discounting rate (*k*), *n* = 22. Analysis yielded a significant main effect of condition on discounting rate (*k*), *F*_(2, 36)_ = 8.98, *p* = 0.001, $\eta_{p}^{2}$ = 0.33. Pairwise comparisons demonstrated that discounting rate (*k*) was significantly greater in the active-anodal condition than in sham condition (*p* = 0.008, *q* = 0.04) (Figure 11).

#### 3.2.2 Impulsivity and motor control measured by Stop-Signal Task

Prior to analyses four outliers were removed for a sample size of *n* = 23. The ANCOVA demonstrated no significant effect for each condition, *F*_(2, 36)_ =1.57, *p* = 0.23.

#### 3.2.3 Cognitive flexibility measured by Uses Task

Separate ANCOVAs were completed for each outcome measure. Technical failures (responses not recorded for some conditions) resulted in listwise deletion of five participants, final sample size of *n* = 22.

For the novelty ratings, there was a violation of sphericity that was corrected for with the use of Greenhouse-Geisser value. There was no effect of condition, *F*_(1.53, 27.45)_ = 0.02, *p* = 0.95. There was no significant effect of stimulation, *F*_(2, 36)_ = 0.03, *p* = 0.97.

#### 3.2.4 Fatigue measured by Karolinska Sleepiness Scale

One participant's data was removed prior to analyses for missing data. The remaining sample size was *n* = 26. The results of the ANCOVA did not support an effect of condition on sleepiness ratings between pre- and post-testing, *F*_(2, 44)_ = 0.20, *p* = 0.82.

#### 3.2.5 Mood measured by Profile of Mood States—Short Form

A MANCOVA included seven subscales as outcome measures: tension, anger, fatigue, depression, confusion, vigor, and esteem-related affect. Missing data was reported for four participants, resulting in a sample size of *n* = 23. There was a significant effect of condition on the fatigue subscale, *F*_(2, 38)_ = 3.83, *p* = 0.031, $\eta_{p}^{2}$ = 0.17. Pairwise comparisons determined fatigue was greater for the sham condition than in the active-cathodal condition. However, one-sample *t*-tests, using zero as the test value, showed fatigue scores increased between pre- and post-testing in the sham condition [*t*_(25)_ = 2.48, *p* < 0.001, *q* = 0.01] whereas fatigue remained stable across both active conditions.

#### 3.2.6 Physical side effects measured by symptom checklist

Self-report data was collected from pre- and post-stimulation for each condition. Based on the checklist, eight symptoms were reported amongst each condition: headache, nausea, scalp irritation, tingling, itching, burning sensation, fatigue, difficulty concentration, and reported pain under electrode(s). Two symptoms were reported being present prior to pre-stimulation and were thus not considered a possible side effect of tDCS application: neck pain and back pain.

All conditions were further assessed by severity (1-absent to 10—severe). The most common reported symptom was tingling (*n* = 11) followed by itching (*n* = 8). The highest rated severity for both of these symptoms was a three. Please refer to Table 4 for the ratings per condition for each symptom. The next most common symptoms reported were a burning sensation (*n* = 5) and difficulty concentrating (*n* = 5). For burning sensation, the highest rating was a three (please refer to Table for remainder ratings). Regarding difficulty concentrating, the highest rating was an eight by one participant. The remaining ratings were below four. The remaining reported symptoms were only reported by one individual: headache, nausea, and fatigue. These all were rated as three and below. There were no reported symptoms for nervousness/anxiety, acute mood change, increased heart rate, blurred vision, hot flush, or dizziness.

**Table 4:** Frequencies of symptoms reported after each stimulation condition.

**Symptom**	**Sham**	**Active-anodal**	**Active-cathodal**
Nervousness or anxiety	0	0	0
Acute mood change	0	0	0
Headache	0	0	1
Severity rating			1
Nausea	1	0	0
Severity rating	3		
Neck pain^*^	0	0	0
Increased heart rate	0	0	0
Back pain^*^	0	0	0
Blurred vision	0	0	0
Scalp irritation	0	2	0
Severity rating	0	2 (*n* = 1)	1
		3 (*n* = 1)	
Tingling	1	5	5
Severity rating	1	2 (*n* = 2)	1 (*n* = 2)
		3 (*n* = 3)	2 (*n* = 3)
Itching	3	3	2
Severity rating	1 (*n* = 1)	1 (*n* = 1)	1 (*n* = 1)
	2 (*n* = 1)	2 (*n* = 1)	2 (*n* = 1)
	3 (*n* = 1)	3 (*n* = 1)	
Burning sensation	0	3	2
Severity rating		1 (*n* = 3)	1 (*n* = 1)
			1 (*n* = 2)
Hot flush	0	0	0
Dizziness	0	0	0
Fatigue	0	1	0
Severity rating	0	1	0
Difficulty concentrating	1	3	1
Severity rating	2	2 (*n* = 2)	4
		8 (*n* = 1)	
Pain under electrode(s)	0	1	1
Severity rating		1	2

^*^Two participants reported symptoms pre-stimulation. Severity scores ranged from 1 (absent) to 10 (severe).

#### 3.2.7 Blinding efficacy

Of the 27 participants, only 12 (44%) correctly indicated when the sham condition occurred. Given this value is below chance, it appears that the blinding procedures were sufficient.

## 4 Discussion

The purpose of the current study was to investigate the effects of tDCS application to the left DLPFC on cognitive performance as well as to note any secondary side effects in relation to mood, impulsivity, risk taking, cognitive flexibility and medically-relevant physical outcomes. This study focused on a healthy, military population, which by nature somewhat limits the generalizability of findings to other populations. Overall, the application of tDCS was associated with enhancement for executive function measured by performance on the Shifting Attention Task, and sustained attention measured by the Rapid Visual Information Processing task.

Regarding executive function, faster reaction times and increased accuracy were found for *both* active-anodal and -cathodal tDCS compared to sham. For sustained attention, there was a main effect between gender and tDCS condition. Faster reaction time and increased *d*′ was noted with active-anodal tDCS compared to both active-cathodal and sham for males only. While with females, there was an increase in both reaction time and *d*′ for active-cathodal compared to active-anodal and sham. Furthermore, these findings were only found during the *fast* mode of the task. Alternatively, assessment of working memory through the Digit Span Task found *decreased* performance with active-cathodal tDCS compared to active-anodal and sham. Study outcomes are summarized in Table 5.

**Table 5:** Summary of study findings.

**Cognitive construct**	**Task**	**Condition(s)**	**Outcome**	**Effect size**
Working memory	Digit span task	Active-cathodal	Worsened performance—lower digit span compared to sham	Large effect size, $\eta_{p}^{2}$ = 0.18
Sustained attention	Rapid Visual Information Processing Task	Active-anodal *and* active-cathodal	Improved performance—Faster reaction time compared to sham *and* active-cathodal Improved d' compared to sham *and* active-cathodal	Overall large main effect, $\eta_{p}^{2}$ = 0.16. For males, large effect for anodal stimulation for reaction time $\eta_{p}^{2}$ = 16. For females, large effect for cathodal stimulation for d' $\eta_{p}^{2}$ = 0.18.
Executive function	Shifting Attention Task	Active-anodal *and* active-cathodal	Improved performance—faster reaction time compared to sham—increased accuracy compared to sham	Large effect for reaction time: $\eta_{p}^{2}$ = 0.15 Large effect for accuracy: $\eta_{p}^{2}$ = 0.16
Risk taking	Delay Discounting Task	Active-anodal	Increased risk taking—higher discounting rate compared to sham	Large effect: $\eta_{p}^{2}$ = 0.33.
Fatigue	Profile of Mood States—Short Form	Active-anodal *and* active-cathodal	Less fatigue—scores increased for sham between pre- and post-stimulation	Large effect: *$\eta_{p}^{2}$* = 0.17

The assessment of side effects showed an increase in impulsivity and risk-taking as measured by the Delay Discounting task when participants received active-anodal stimulation compared to sham. However, this was not replicated in the Stop Signal Task, which is another measure of impulsivity, but one that focuses more on motor control rather than risk-taking behaviors. No differences were found in cognitive flexibility (as measured by the Uses Task). The only change in self-reported fatigue was noted in the Profile of Moods State-Short Form (POMS-SF) ratings [as opposed to the Karolinska Sleepiness Scale (KSS)]. Here, participants reported an increase in fatigue from pre-stimulation to post-stimulation in the sham condition only compared while both active-anodal and -cathodal remained stable. Finally, some physical symptoms as a result of stimulation were reported, with tingling and itching being the most common sensations, although characterized by low severity ratings.

The findings we report here are in-line with the variety of findings within the literature, thus possibly helping to clear up some confusion and perhaps add to it. Interestingly, our measure of executive function through the Shifting Attention Task, yielded performance improvements in *both* active-anodal and -cathodal tDCS with both resulting in faster reaction times and higher accuracy rates. Notably, this task utilizes multiple cognitive functions, such as set shifting and attention. Improvement on this task may suggest that improved performance can be found with both the excitatory processes resulting from anodal-tDCS and the inhibitory processes from cathodal-tDCS, or co-stimulation in peripheral nerves may be influencing cognitive activation (Madji et al., 2023). However, this is simply speculation, thus our findings add to the disparities in the current literature regarding consistent findings. Furthermore, our findings related to sustained attention, with active-anodal improving reaction times during the fast mode only, provides possible insight into the conditions under which tDCS may be most effective. This may indicate performance enhancement from anodal stimulation may only be noticeable with higher “workload,” or more difficult, task conditions. Similar results have been demonstrated where Nelson et al. (2019) found active-anodal tDCS applied to the same region (left DLPFC, at 2 mA) resulted in performance improvements compared to sham on a multitasking paradigm that is characterized by a high workload. Thus, this may add to determining when or under what conditions tDCS is most effective. The fact that we found no improvements on working memory with our chosen parameters seems to be in-line with literature reviews on the topic (Müller et al., 2022; Senkowski et al., 2022). For example, decreased performance with active-cathodal tDCS on the Digit Span Task has been previously found (Boehringer et al., 2013). Thus, again supporting the idea that the directionality of the current and the brain regions targeted, can differentially influence the various performance outcomes. However, it should also be noted that due to our time constraints, we included lower numbers of trials than is typically used on some of these tasks, such as the Stroop task. This may have limited the detection of true effects. Moreover, having participants engaged in tasks targeting different cognitive functions while receiving tDCS may have impacted outcomes, and potentially canceled out potential task improvements (e.g., Li et al., 2019). Part of the motivation for including multiple types of tasks was that many of the tasks military members engage in draw from several different cognitive processes (Feltman et al., 2021). Thus, we were interested in how application to one location might impact those. However, to gain a clearer understanding of the effects of tDCS on these distinct cognitive processes, individual study of each is needed.

Concerning the various side effects assessed, the increase in discounting rate, measured by the Delay Discounting task, with the application of active-anodal tDCS, is of greatest interest. This task measures impulsivity and risk taking through the presentation of a series of monetary rewards that are either available “now” or at some later date (e.g., 8 weeks from now). Participants are presented with a variety of choices, such as to receive a lower amount now, or wait some period for a higher amount. The discounting rate gives an indication of how they selected those reward options. A higher rate is indicative of someone discounting larger future rewards and selecting the immediate amount, thus potentially being more impulsive and likely to take risks, though, the relationship between the outcomes of the task and real-world impulsivity is controversial (Bailey et al., 2021). As such, for healthy populations, this is a performance tradeoff (i.e., potential to improve attention at the cost of increased impulsivity and risk taking) that may be undesirable, especially with persons in critical occupations roles, such as the military where impulsive and risky behaviors can have dire consequences. Indeed, this task was chosen to gain a holistic picture of the types of effects tDCS has on cognitive processes beyond those that are targeted. Increased risk-taking can be of considerable concern for a military population. Nonetheless, evidence within the neuroscientific literature supports that our stimulation of the prefrontal region may have impacted this change, given that the frontal region (along with parietal and posterior cingulate cortex) has been found to play a role in determining the value of delay rewards (Peters and Büchel, 2009). Of course, further study is needed to support and better understand this finding.

There were no reported changes in mood besides fatigue as reported by the Profile of POMS-SF questionnaire. Fatigue was significantly greater for the sham as compared to active-anodal and -cathodal conditions. Post-stimulation fatigue ratings within the POMS-SF significantly increased during sham compared to pre-stimulation but remained stable during both active stimulation sessions. However, our other fatigue measure, the KSS, did not corroborate these findings and showed no significant differences pre- and post-stimulation for any of the conditions. Thus, it is possible each of the active stimulation conditions played a role in mitigating the onset of fatigue, which has been shown in previous literature (Motohashi et al., 2013). The difference in fatigue findings is likely related to how each of these questionnaires measure fatigue. The KSS focuses solely on sleepiness, whereas the POMS-SF asks participants to rate how they feel *right now* across a variety of descriptor words. The fatigue subscale words include the following: worn out; fatigued; exhausted; weary; and bushed. Thus, the POMS-SF is likely getting at different types of fatigue rather than just sleepiness. Thus, the application of tDCS, whether anodal or cathodal, may be impacting an aspect of fatigue beyond drowsiness.

The lack of any other reported mood side effects is consistent with previous literature in healthy populations (Plazier et al., 2012; Motohashi et al., 2013). Physical symptoms reported most were itching, tingling, scalp irritation, and burning sensation. Though perceptions of itchiness, pain and irritation may be difficult to distinguish. The symptom checklist thus may have some overlapping reportable symptoms. In addition, difficulty concentrating was also reported by all individuals in each stimulation condition. These symptoms are all commonly reported after single session tDCS application in addition to complaints of headache and fatigue (Eryilmaz et al., 2014; Rassovsky et al., 2018). As previously mentioned, side effects need to be considered as trade-offs for cognitive performance enhancement in certain high-risk populations such as the military.

Our study is not without limitations. One such limitation is that our data collection was interrupted by the COVID-19 pandemic. Approximately half of the participants completed the study after data collection resumed in October 2020. When data collection was able to resume, several safety measures were put in place, such as mask-wearing by participants and data-collectors. It is possible that this impacted outcomes, however, we have not seen any literature to-date to support this. An additional limitation is that we did not correct for multiple comparisons. We concluded that a Bonferroni correction would be too conservation in this situation due to not having a large amount of multiple statistical comparisons, and this decision helped to reduce risk of a type II error (Armstrong, 2014). Additionally, as was previously mentioned, there was a time-constraint enacted on some of the tasks, resulting in some tasks having a lower trial number than is typically used. Another limitation of this study that warrants attention involves its design, where tDCS stimulation occurred during the completion of multiple cognitive tasks conducted in one session. The completion of the cognitive tasks began ~5 min after tDCS stimulation started, which has potential implications for the effects of the stimulation. Due to tDCS preferentially affecting neurons that are already engaged in the current task being completed, the effects of tDCS across multiple tasks may risk having split effects between two tasks, or the performance in one task may cancel out the other (Fertonani and Miniussi, 2017; Kronberg et al., 2019; Boroda et al., 2020). Though, in the current study multiple tasks were completed and the order of the tasks during each session was randomized, this is still worth noting as a limitation. Future research should consider separating out tasks by 5 min or longer, or simply consider avoiding multiple tasks of different cognitive processes during the same tDCS stimulation session. Additionally, future research should examine the exploration of other differences in parameters for tDCS application, such as strength of current, length of time, location of electrode placements, repeated sessions, and individual differences (i.e., age, hormones) (Krause and Kadosh, 2014; Agboada et al., 2019; Feltman et al., 2020). For a more comprehensive list, a recent systematic review done by Santander et al. (2024) goes into further detail regarding various outcomes for the numerous methodological parameters used in tDCS research. For example, different strengths can result in various outcomes on performance and transference to additional tasks and is not always associated with improved performances (Krause and Kadosh, 2014; Ehrhardt et al., 2021). Finally, the three tasks to measure potential behavioral side effects were all completed ~1-h post-stimulation (due to completion of military tasks immediately following cognitive tasks and stimulation). As such, it is possible that some behavioral changes were not captured, as the stimulation may have “worn off.”

Results of this study provide additional information regarding the selection of stimulation parameters for performance enhancement in healthy individuals along with some practical implications in terms of “real-world” application. Specifically, we found that active-anodal to the left DLPFC at 2 mA, for 30 min, improved sustained attention under high workload and executive function (for each reaction time and accuracy). However, this improvement may be at the cost of increased risk taking and impulsivity. For personnel in high-stakes occupations, such as military members, the potential for such a side effect may not be worth the potential for improvements in other aspects of cognition. Alternatively, active-cathodal tDCS, using the same parameters, was found to also improve executive function for each reaction time and accuracy, but may come at the cost of decreased working memory. Neither stimulation mode (anodal or cathodal) presented serious adverse effects that would warrant not recommending pursuit of either. Rather, these results suggest that to truly understand how tDCS performs in the “real-world” it may be beneficial to use “real-world” tasks that draw on these multiple cognitive processes. In doing so, researchers may be able to more confidently identify the stimulation parameters that are most beneficial for said “real-world” tasks. In addition, future research should build off our findings here to further explore the various secondary effects that tDCS has on performance. While we demonstrated initial findings related to the potential performance tradeoff involving changes to risk taking behavior, these types of evaluations should be built into standard tDCS protocols to gain a holistic depiction of how tDCS impacts cognition.

## Data availability statement

Data are not available per institutional policy. However, data can be shared by establishing a data sharing agreement. Please contact KF at kathryn.a.feltman.civ@health.mil for data requests.

## Ethics statement

This research involved human subjects and was approved by the U.S. Army Medical Research and Development Command's Office of Research Protections Institutional Review Board. This research was conducted in accordance with the local legislation and institutional requirements. All participants provided their written informed consent prior to participating in the study.

## Author contributions

MD: Writing – original draft, Writing – review & editing. KF: Investigation, Methodology, Writing – original draft, Writing – review & editing. AK: Conceptualization, Formal analysis, Writing – original draft, Writing – review & editing. RM: Data curation, Formal analysis, Writing – original draft, Writing – review & editing.
