# Supplementary material for: Limitations associated with transcranial direct current stimulation for enhancement: considerations of performance tradeoffs in active-duty Soldiers
Source: Front Hum Neurosci. 2024 Jul 26;18:1444450. doi: 10.3389/fnhum.2024.1444450 (PMC11310018; doi:10.3389/fnhum.2024.1444450)

Version date: 29 August 2019

Subject: ________ Test Session: ______ Research Team Member's Initials: _____

SYMPTOM CHECKLIST

INSTRUCTIONS TO SUBJECTS: "I am going to ask you if you are **CURRENTLY** experiencing any of the following symptoms. If you are, I'll ask you to rate its severity using this scale [show picture of severity scale], and tell me when you first noticed it."

If exact time onset is unknown, try to estimate onset within a 15-minute range based on study events.

TECHNICIANS: Verify that subject's experience is **CURRENT** (i.e., happening RIGHT NOW)! If subject reports that the symptom was experienced PREVIOUSLY, do NOT mark it on this sheet – mark it on EXIT PHYSICAL EXAM sheet.

ITEM	Y / N (circle one)	IF "YES" CIRCLE ONE Using severity scale	Time of Onset
**Nervousness or Anxiety**	**Y / N**	1 2 3 4 5 6 7 8 9 10	
**Acute mood change**	**Y / N**	1 2 3 4 5 6 7 8 9 10	
**Headache**	**Y / N**	1 2 3 4 5 6 7 8 9 10	
**Nausea**	**Y / N**	1 2 3 4 5 6 7 8 9 10	
**Neck pain**	**Y / N**	1 2 3 4 5 6 7 8 9 10	
**Increased heart rate**	**Y / N**	1 2 3 4 5 6 7 8 9 10	
**Back pain**	**Y / N**	1 2 3 4 5 6 7 8 9 10	
**Blurred vision**	**Y / N**	1 2 3 4 5 6 7 8 9 10	
**Scalp irritation**	**Y / N**	1 2 3 4 5 6 7 8 9 10	
**Tingling**	**Y / N**	1 2 3 4 5 6 7 8 9 10	
**Itching**	**Y / N**	1 2 3 4 5 6 7 8 9 10	
**Burning sensation**	**Y / N**	1 2 3 4 5 6 7 8 9 10	
**Hot flush**	**Y / N**	1 2 3 4 5 6 7 8 9 10	
**Dizziness**	**Y / N**	1 2 3 4 5 6 7 8 9 10	
**Fatigue**	**Y / N**	1 2 3 4 5 6 7 8 9 10	
**Pain under electrode(s)**	**Y / N**	1 2 3 4 5 6 7 8 9 10	
**Difficulty concentrating**	**Y / N**	1 2 3 4 5 6 7 8 9 10	

"Do you have anything else going on RIGHT NOW that I haven't asked you about?"

If yes, ask subject to describe symptom or event. Write down their description VERBATIM.
Ask subject to rate severity and give time of onset.

**ITEM**	**Y / N (circle one)**	**IF "YES" CIRCLE ONE**	**Time of Onset**
	**Y / N**	1 2 3 4 5 6 7 8 9 10	
	**Y / N**	1 2 3 4 5 6 7 8 9 10	
	**Y / N**	1 2 3 4 5 6 7 8 9 10	
	**Y / N**	1 2 3 4 5 6 7 8 9 10	

Vital sign measurements to be recorded by research team member.

**Temperature**	**Blood Pressure**	**Respiration Rate**	**Pulse Rate**	**Oxygen Saturation**

Instructions for application

If participants have scored '5' or above for any of the statements*, they should stay in the laboratory until symptoms have subsided or until they and the researcher are satisfied for them to leave. If their symptoms persist for more than 24 hours then the researcher should be contacted and appropriate medical attention should be sought.

*Apart from the following pseudo items: back pain, increased heart rate, hot flash, and dizziness

Severity Scale to be presented to subject

1	2	3	4	5	6	7	8	9	10

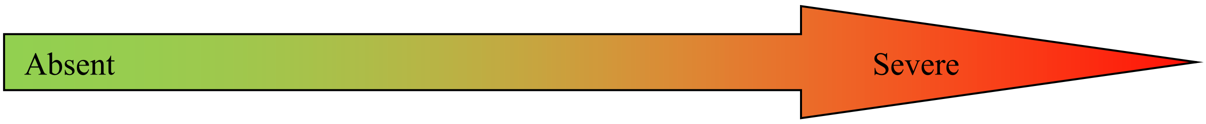

Supplement: Supplementary file 1 [file Data_Sheet_1.PDF]
