# Supplementary material for: Limitations associated with transcranial direct current stimulation for enhancement: considerations of performance tradeoffs in active-duty Soldiers
Source: Front Hum Neurosci. 2024 Jul 26;18:1444450. doi: 10.3389/fnhum.2024.1444450 (PMC11310018; doi:10.3389/fnhum.2024.1444450)
Supplement: Supplementary file 2 [file Data_Sheet_2.pdf]

Version date: 29 August 2019

PHYSICIAN SCREENING CHECK LIST

TODAY'S DATE ______/______/______
YOUR INITIALS (Study Physician) ________

SUBJECT NUMBER ____________

Are you on active military duty? .... **YES** **NO**

Are you National Guard or Reservist? …………….. **YES** **NO**

If yes, circle which………………………………… **NG** **Reserve**

I am going to ask you a series of questions. For your own safety, please answer all of the questions honestly and to the best of your knowledge. Let me know if you don't understand a question.

DATE OF BIRTH ________/________/________
day month year

AGE .... ___________ **(must be 19-40 years of age)**
HEIGHT .... ___________ ft/inches (or cm)
WEIGHT .... ___________ lbs (or kg)
RIGHT or LEFT-HANDED? .... **RIGHT** **LEFT** **BOTH**

DEPENDENCE HISTORY

Have you ever been diagnosed with a psychiatric illness? **Yes** **No**
____

Have you ever been hospitalized for a psychiatric or psychological reason? **Yes** **No**
____

Have you ever used or abused illegal or prescription drugs? **Yes** **No**
____

Have you ever been under medical care for an addiction of any kind? **Yes** **No**
____

Disqualify if subject has a history of drug abuse or history of addiction.

ALCOHOL USE

On average, how many beers do you drink per week? ___________
On average, how many glasses of wine do you drink per week? ___________
On average, how many mixed drinks or shots do you drink per week? ___________
Disqualify if more than 6 beers/week or 4 glasses wine or 4 mixed drinks/week

NICOTINE USE

Do you use products containing nicotine? .... YES NO
– including e-cigarettes, patches/gum, cigars
If Yes, what? ____________________________

On average, how many times per week do use _______? ___________
On average, how long before you experience withdrawal symptoms? ___________

MEDICATION USE

Are you currently taking any prescription drugs? .... YES NO
If Yes, what? _____________________________
For what? _____________________________

Are you currently taking any over-the-counter drugs? .... YES NO
If Yes, what? _____________________________
For what?_____________________________

Are you currently taking any vitamins or dietary supplements? .... YES NO
If Yes, what? _____________________________
For what?_____________________________

Study Physician will determine whether any of the above is exclusionary

Version date: 29 August 2019

MEDICAL HISTORY

Have you ever had, or do you now have: (Y=YES N=NO D=DON'T KNOW)

IF YES, obtain (1) Type; (2) Mo/Yr of occurrence; (3) Is it current?

Y N D .... Attention Deficit Disorder?

____

Y N D .... Neurocognitive Disorders (e.g., cognitive disabilities)?

____

Y N D .... Psychiatric Disorders (e.g., depression, anxiety, bipolar)?

____

Y N D .... Head injury?

____

Y N D .... Loss of consciousness, including fainting or passing out?

____

Y N D .... Asthma?

____

Y N D .... Have you ever had a seizure?

____

Y N D .... Do you suffer from migraines?

____

Y N D .... Do you have any metal in your head (other than inside the mouth) such as shrapnel or surgical clips?

____

Y N D .... Do you have any implanted devices (e.g., cardiac pacemaker, brain stimulator)?

____

Y N D .... Head wound that has not completely healed?

____

Y N D .... Neuritis?

____

Y N D .... Eye trouble?

____

Y N D .... Do you wear glasses or contacts?

____

Y N D .... Ever had an adverse reaction to tDCS or any other brain stimulation technique (e.g., TMS, tRNS)?

____

Y N D .... Bled excessively after injury or tooth extraction?

____

Y N D .... Skin diseases, particularly on your scalp?

____

Y N D .... Coughed up blood?

____

Y N D .... Shortness of breath?

____

Y N D .... Pain or Pressure in the chest? (circle which)

____

Y N D .... Rapid or Pounding heartbeat? (circle which)

____

Y N D .... High or Low blood pressure? (circle which)

____

Y N D .... Any condition that may interfere with placing the reference electrode on your upper right arm? Such as a recent injury or metal (e.g., shrapnel, surgical clips)?

____

Y N D .... Bad reaction to drugs, medicines, or serum? (circle which)

____

Y N D .... Have you ever been hospitalized for injury or illness?
If YES, what/when?

____

what/when?

____

what/when?

____

Study Physician will determine whether any of the above is exclusionary (check whether cleared for study or did not qualify and sign.

____________	____________	____
Cleared	***DNQ***	***Physician Signature & Date***
