# Supplementary material for: Limitations associated with transcranial direct current stimulation for enhancement: considerations of performance tradeoffs in active-duty Soldiers
Source: Front Hum Neurosci. 2024 Jul 26;18:1444450. doi: 10.3389/fnhum.2024.1444450 (PMC11310018; doi:10.3389/fnhum.2024.1444450)
Supplement: Supplementary file 3 [file Data_Sheet_3.pdf]

Version Date: 20 Dec 2018

Subject ID: _____________ Test session: _________ Research Team Member Initials: ________

Post Study Questionnaire

Please select a response that most accurately reflects your agreement with the statements below.

1. I could easily learn how to use tDCS.

   ___ Strongly Disagree

   ___ Disagree

   ___ Undecided

   ___ Agree

   ___ Strongly Agree

2. It is a good idea for soldiers to use tDCS as a cognitive enhancement tool in operational environments:

   ___ Strongly Disagree

   ___ Disagree

   ___ Undecided

   ___ Agree

   ___ Strongly Agree

3. Most of my fellow soldiers will welcome the fact that I use tDCS as a cognitive enhancement tool.

   ___ Strongly Disagree

   ___ Disagree

   ___ Undecided

   ___ Agree

   ___ Strongly Agree

4. The military will encourage the use of tDCS as a cognitive enhancement tool:

    ___ Strongly Disagree

    ___ Disagree

    ___ Undecided

    ___ Agree

    ___ Strongly Agree

5. I feel comfortable with using tDCS in an operational environment:

    ___ Strongly Disagree

    ___ Disagree

    ___ Undecided

    ___ Agree

    ___ Strongly Agree

6. I intend to use tDCS as a cognitive enhancement tool in operational settings if/when the military makes it available to soldiers.

    ___ Strongly Disagree

    ___ Disagree

    ___ Undecided

    ___ Agree

    ___ Strongly Agree
