# Supplementary figures and images for: Limitations associated with transcranial direct current stimulation for enhancement: considerations of performance tradeoffs in active-duty Soldiers
Source: Front Hum Neurosci. 2024 Jul 26;18:1444450. doi: 10.3389/fnhum.2024.1444450 (PMC11310018; doi:10.3389/fnhum.2024.1444450)

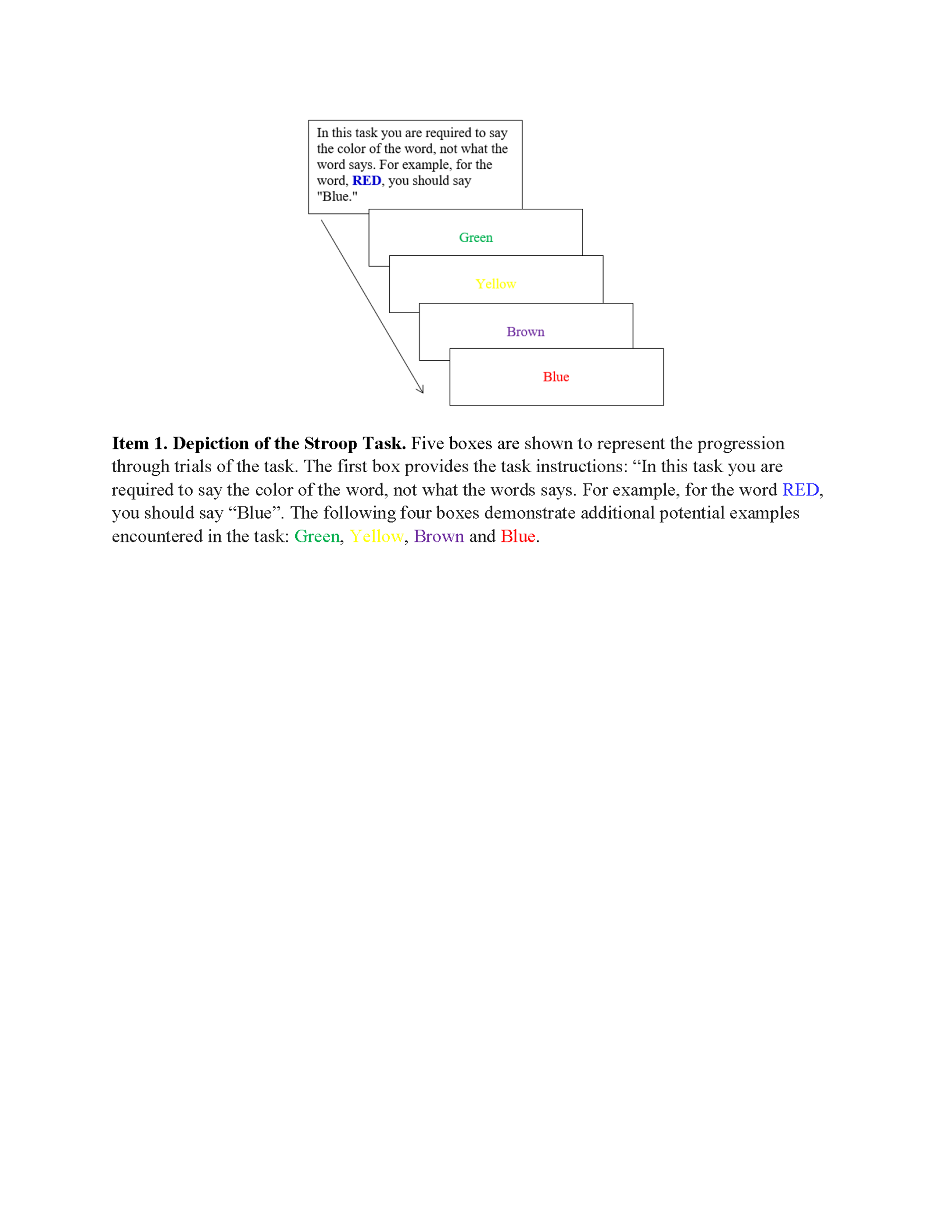

Supplement: Supplementary file 4 [file Image_1.tif]

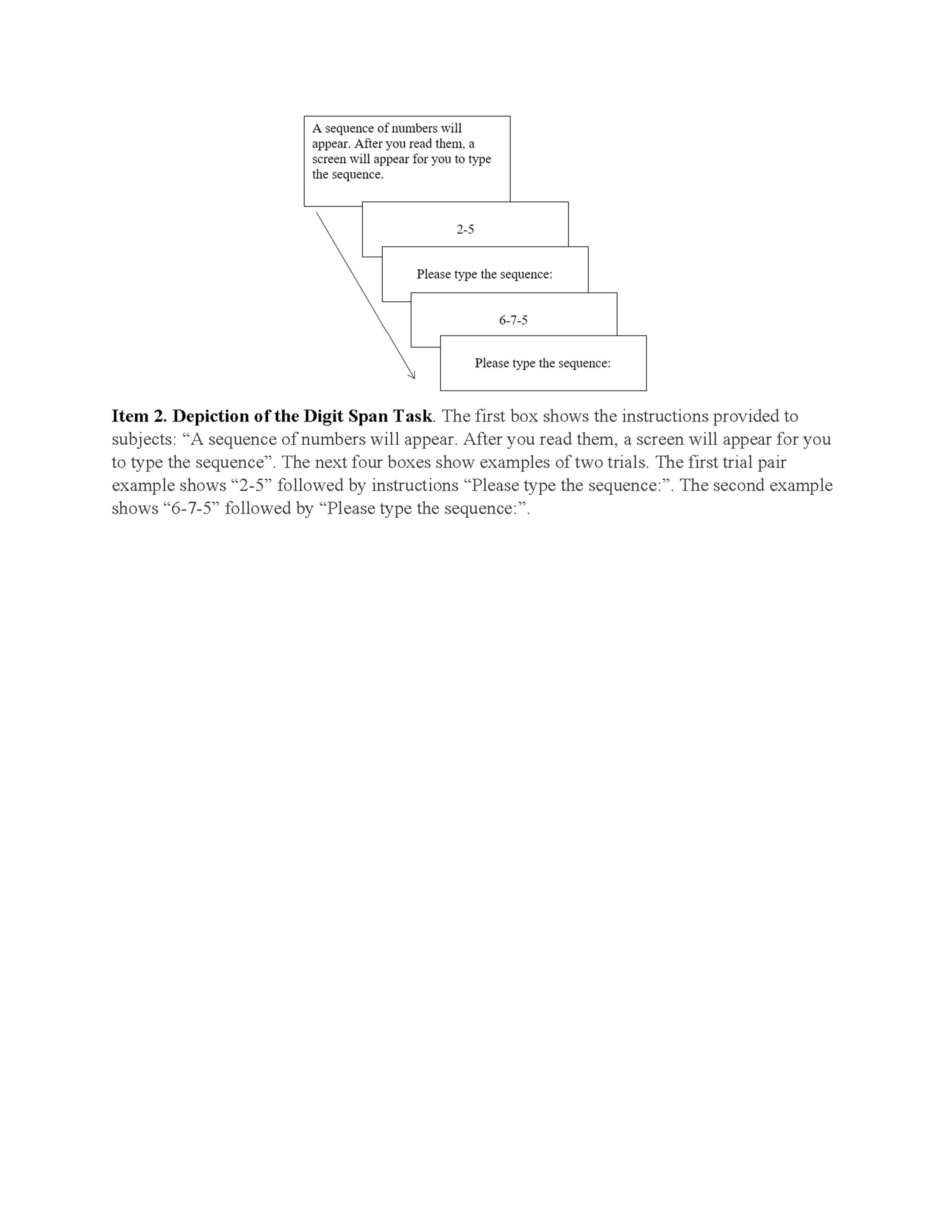

Supplement: Supplementary file 5 [file Image_2.tif]

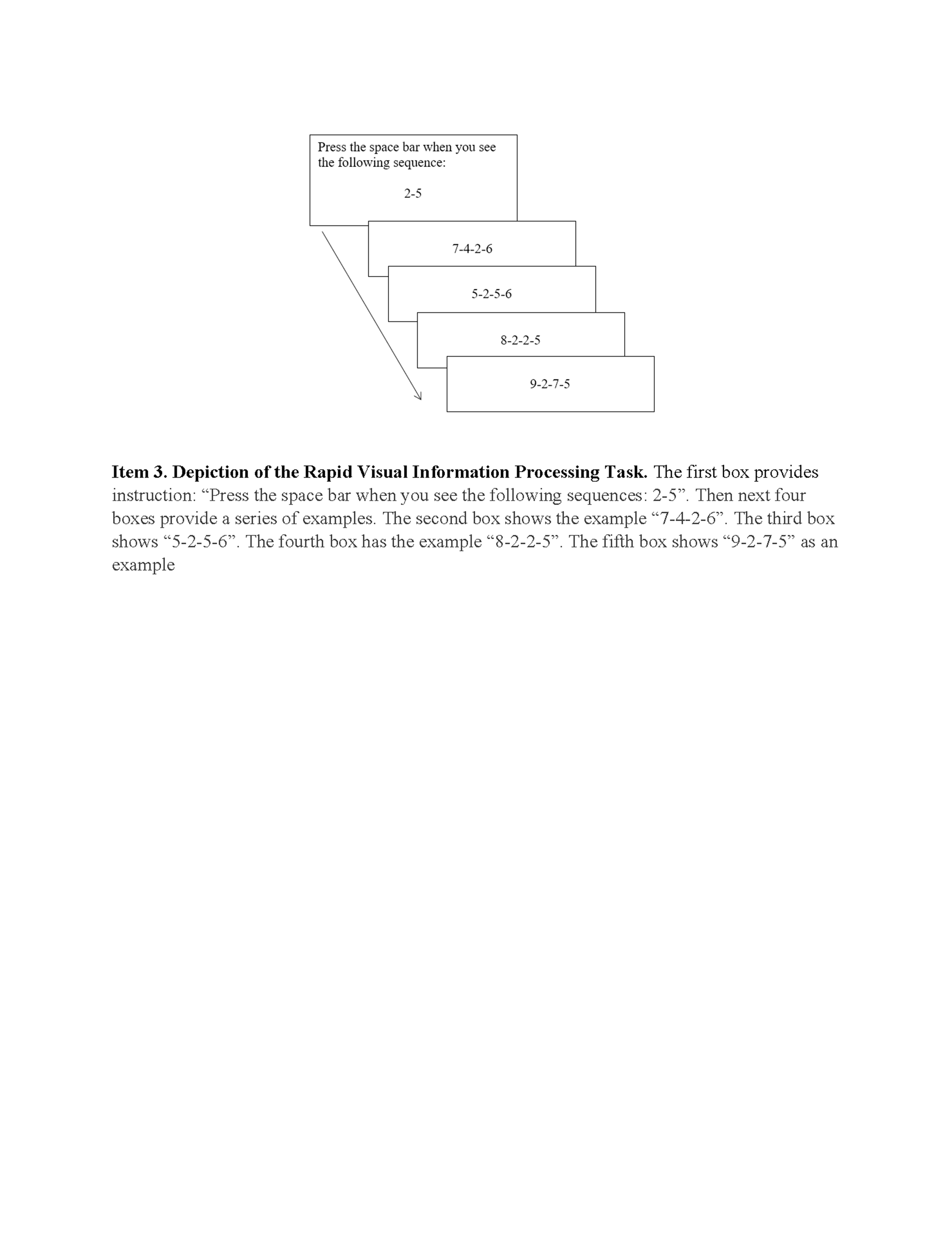

Supplement: Supplementary file 6 [file Image_3.tif]

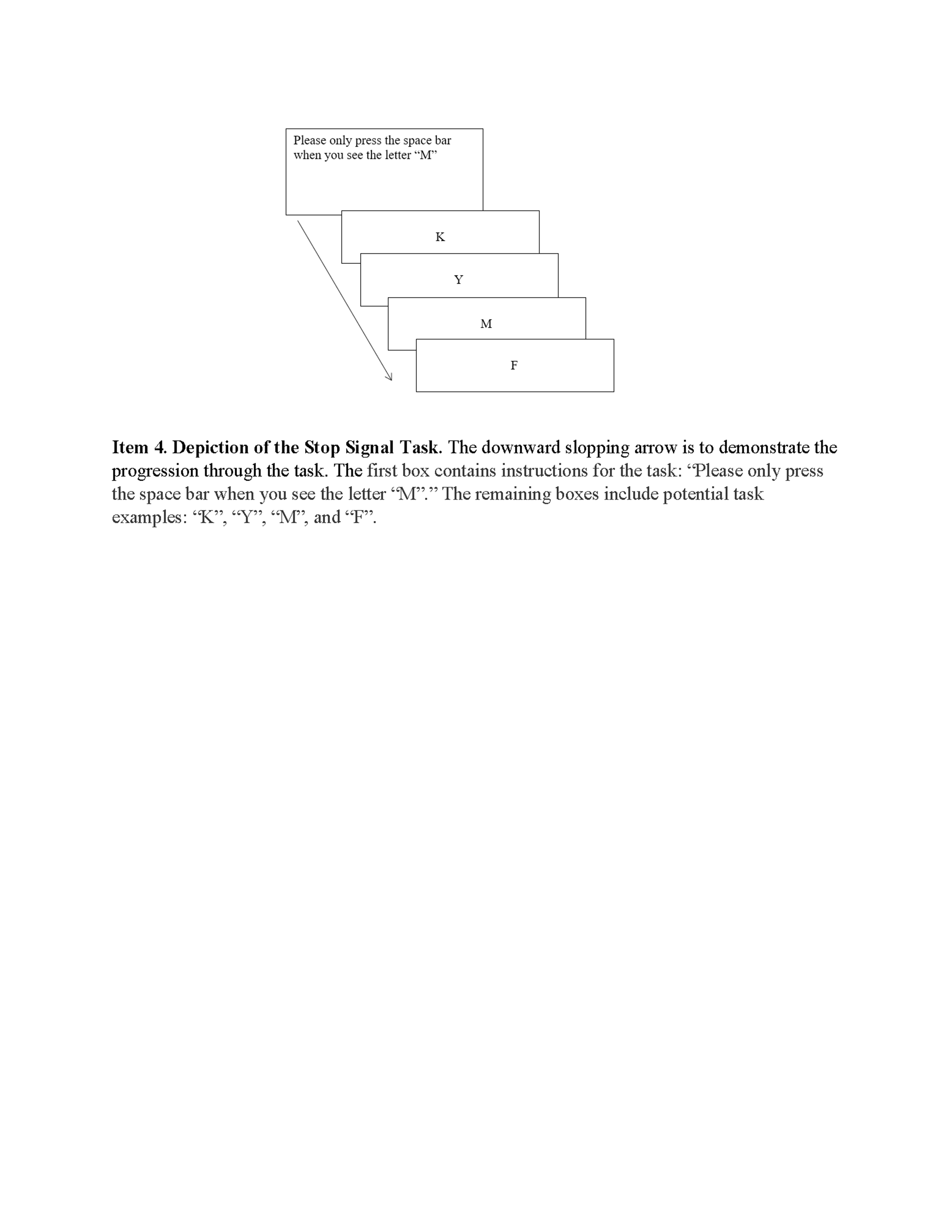

Supplement: Supplementary file 7 [file Image_4.tif]

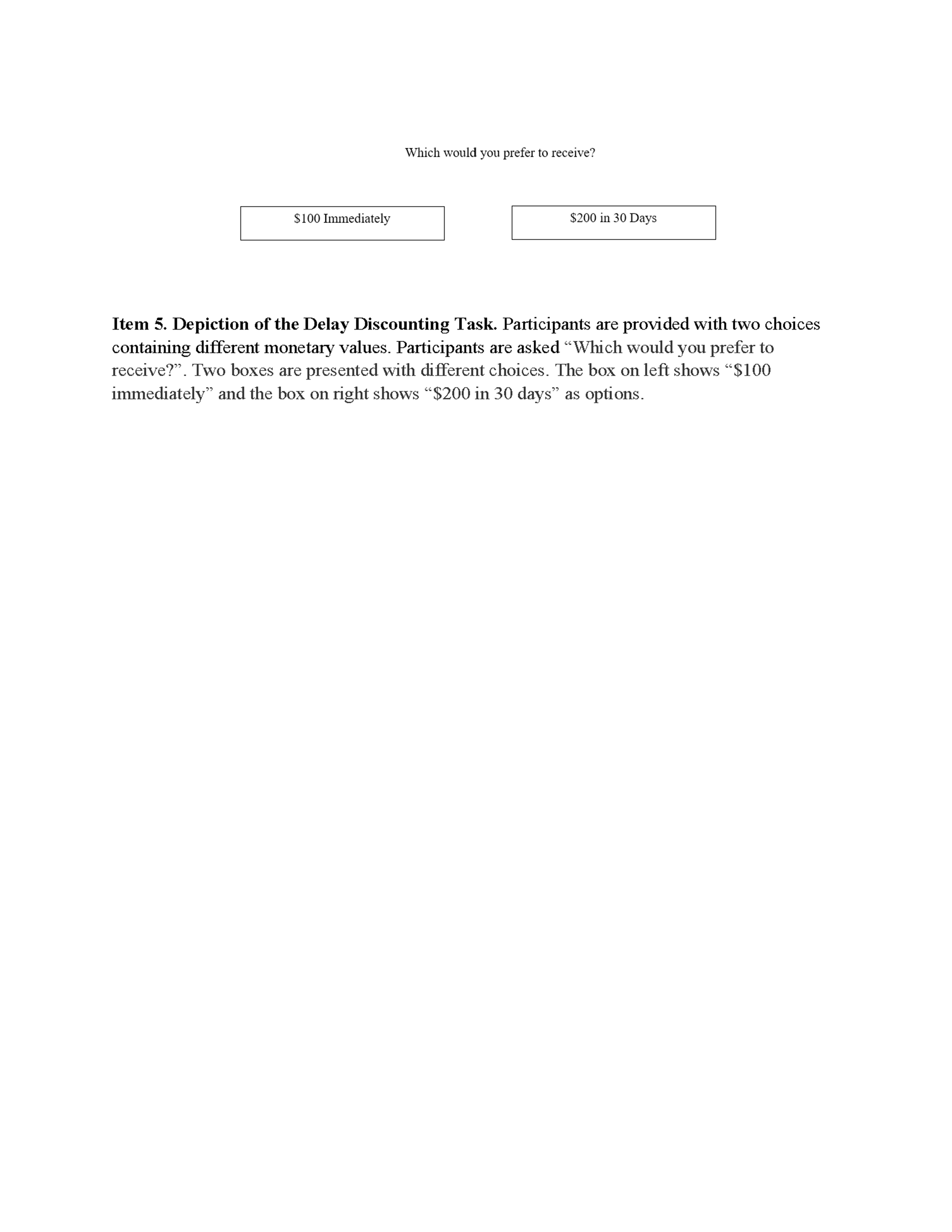

Supplement: Supplementary file 8 [file Image_5.tif]

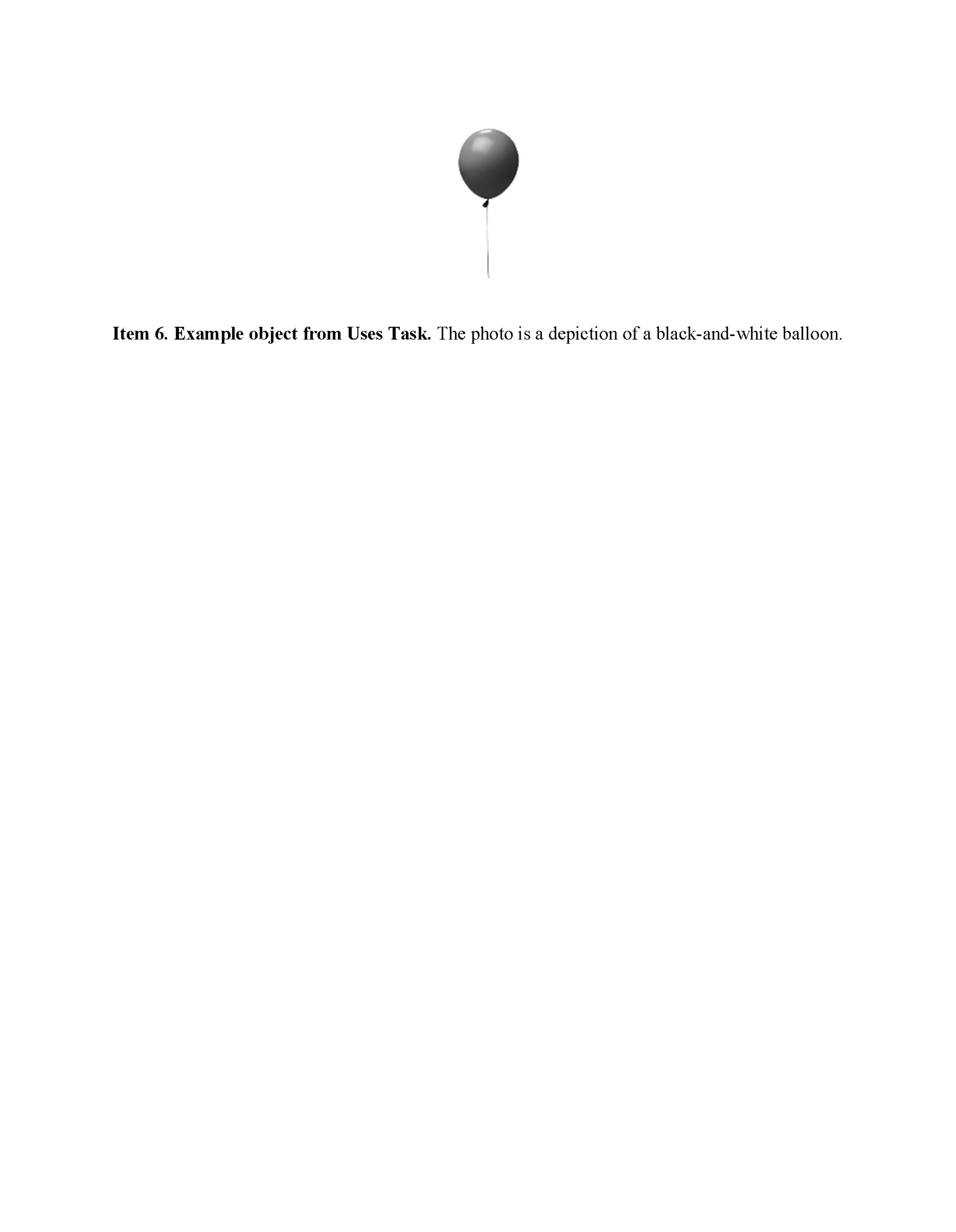

Supplement: Supplementary file 9 [file Image_6.tif]
